# Identifying and quantifying radiation damage at the atomic level

**DOI:** 10.1107/S1600577515002131

**Published:** 2015-02-14

**Authors:** Markus Gerstel, Charlotte M. Deane, Elspeth F. Garman

**Affiliations:** a Laboratory of Molecular Biophysics, Department of Biochemistry, University of Oxford, South Parks Road, Oxford OX1 3QU, UK; b Department of Statistics, University of Oxford, 1 South Parks Road, Oxford OX1 3TG, UK

**Keywords:** radiation damage, specific damage, preferential damage, atomic *B* factors, atomic displacement parameters

## Abstract

Radiation damage impedes macromolecular diffraction experiments. Alongside the well known effects of global radiation damage, site-specific radiation damage affects data quality and the veracity of biological conclusions on protein mechanism and function. Site-specific radiation damage follows a relatively predetermined pattern, in that different structural motifs are affected at different dose regimes: in metal-free proteins, disulfide bonds tend to break first followed by the decarboxylation of aspartic and glutamic acids. Even within these damage motifs the decay does not progress uniformly at equal rates. Within the same protein, radiation-induced electron density decay of a particular chemical group is faster than for the same group elsewhere in the protein: an effect known as preferential specific damage. Here, *B*_Damage_, a new atomic metric, is defined and validated to recognize protein regions susceptible to specific damage and to quantify the damage at these sites. By applying *B*_Damage_ to a large set of known protein structures in a statistical survey, correlations between the rates of damage and various physicochemical parameters were identified. Results indicate that specific radiation damage is independent of secondary protein structure. Different disulfide bond groups (spiral, hook, and staple) show dissimilar radiation damage susceptibility. There is a consistent positive correlation between specific damage and solvent accessibility.

## Abstract 2

A metric indicating the relative level of specific radiation damage for individual atoms, that can be calculated from refined and deposited protein structure models, is presented.

## 1. Introduction   

Radiation damage is an integral part of macromolecular X-ray crystallography (MX). The rate of radiation damage can be reduced, for example by conducting experiments at cryo-temperatures or using scavengers, but not avoided. Using software tools such as *BEST* (Bourenkov & Popov, 2010 ▶) and *RADDOSE-3D* (Zeldin *et al.*, 2013 ▶), data collection strategies can be optimized to minimize the dose absorbed by the available crystal volume. However, in many protein crystallography structure determinations some radiation damage effects will still be evident. There are two types of radiation damage. Global damage can be observed in the decay of the diffraction pattern, and an increase in unit-cell volume and often of mosaicity. The increase in unit-cell volume leads to non-isomorphism and causes difficulties in, for example, MAD (multi-wavelength anomalous dispersion) structure determination. Meanwhile specific radiation damage manifests itself as a change in the electron density around particular protein regions in a repeatable pattern. At 100 K metallo-centres are reduced (Yano *et al.*, 2005 ▶) and disulfide bonds are radicalized (Sutton *et al.*, 2013 ▶) very early in the experiment. Then, disulfide bonds are elongated and broken (Burmeister, 2000 ▶; Ravelli & McSweeney, 2000 ▶; Weik *et al.*, 2000 ▶, 2002 ▶), glutamates and aspartates are decarboxylated (Burmeister, 2000 ▶; Ravelli & McSweeney, 2000 ▶; Weik *et al.*, 2000 ▶; Fioravanti *et al.*, 2007 ▶), tyrosine –OH groups are lost (Burmeister, 2000 ▶), the methionine Sδ—C∊ bond is cleaved, and covalent metal-bonds are broken (Ennifar *et al.*, 2002 ▶; Ramagopal *et al.*, 2005 ▶).

The impact of specific radiation damage goes beyond these effects on isolated residues. Radiation-induced structural changes may cause the experimenter to draw incorrect conclusions from the data. For instance, in the investigation of reaction pathways of functional proteins, structural changes caused by radiation damage may be mistaken as the formation of reaction intermediates, as noted by Matsui *et al.* (2002 ▶) in a study of bacteriorhodopsin. Work on photoactive yellow protein by Kort *et al.* (2004 ▶) identified a new low-temperature photo-intermediate that was only detectable by minimum dose data collection and correction for structural changes induced by specific radiation damage, although structural data were available to 0.85 Å resolution (Genick *et al.*, 1998 ▶).

Specific radiation damage has been shown to depend on a number of different experimental factors. Two studies on the effect of X-ray photon energy on specific damage came to different conclusions: Shimizu *et al.* (2007 ▶) reported no change in specific damage, as determined by atomic *B*-factors (also known as atomic displacement parameters, or *B*-factors for short), at nine different X-ray energies between 6.3 and 33.0 keV. However, Homer *et al.* (2011 ▶) compared specific damage observing real space electron density and reported lower specific damage at disulfide sulfurs with an incident photon energy of 9 keV compared with that at 14 keV, but no change at methionine sulfurs. Both studies were conducted using lysozyme crystals at cryo-temperatures (100 K). The variation in radiation damage susceptibility of the different chemical groups is believed to be due to their different affinity for the secondary electrons which are mobile even at 77 K (Jones *et al.*, 1987 ▶). Disulfide bonds are most susceptible, as they are the most electron affinic part of the protein. Petrova *et al.* (2010 ▶) measured specific damage using a decrease in atomic occupancy as a metric, and found the appearance of new disulfide cysteine rotamers in elastase crystals to be positively correlated with local solvent accessibility at temperatures of both 15 K and 100 K.

The susceptibility of the three disulfide bridges in cubic insulin was investigated by Meents *et al.* (2010 ▶), who showed that the rate of damage to the solvent-exposed disulfide bridge was temperature-dependent, with a fourfold increase in occupancy decay rate at 100 K compared with that at 50 K. The susceptibility of the other two disulfide bridges buried inside the protein showed only a very small temperature dependence. Using atomic *B*-factors, Juers & Weik (2011 ▶) identified a correlation between specific damage and the distance to the nearest solvent channel in thermolysin at 160 K, but did not observe this at 100 K. A similar effect was reported by Warkentin *et al.* (2012 ▶), who, again using atomic *B*-factors, identified seven solvent-exposed turns of thaumatin as being the most radiation-sensitive parts at experimental temperatures of 180 K and above, but not at 155 K and below. Fioravanti *et al.* (2007 ▶) showed that radiation susceptibility does not correlate with solvent accessibility at 100 K for malate dehydrogenase, which, as a halophilic enzyme, contains 35 aspartic and 27 glutamic acid residues in each chain of 304 residues but has no disulfide bonds. Susceptibility was identified by peaks in the 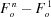 difference Fourier maps between the first and one of the two subsequent datasets, obtained after X-ray burn phases. Homer *et al.* (2011 ▶) also could not find evidence for a relationship between side-chain solvent accessibility and radiation susceptibility, as determined by the electron density decay of lysozyme crystals at 100 K. Some observations indicate that acidic residues with higher p*K*_a_ are more sensitive to radiation damage, but such a correlation was not found by Fioravanti *et al.* (2007 ▶). Ravelli & McSweeney (2000 ▶) could not observe a clear correlation between the susceptibility of aspartic and glutamic acid residues, and either solvent accessibility or p*K*_a_ using lysozyme crystals at 100 K.

To compare observations concerning radiation-induced structural damage between experiments, the decrease in electron density is routinely measured against the absorbed dose (absorbed energy per unit mass; 1 Gy = 1 J kg^−1^). While refined protein structure models from X-ray determinations are now deposited in the Protein Data Bank (PDB; Berman *et al.*, 2003 ▶) together with the corresponding structure factors, they are not usually accompanied by dose values. Therefore users of these protein structure models have generally little to no information available on the degree of specific radiation damage suffered. One reason for this is that it is impossible for the experimenter to directly and quantitatively measure the effective dose absorbed by a crystal. Experimental proxies such as relative isotropic *B*-factors (*B*_rel_) (Kmetko *et al.*, 2006 ▶) or the ratio of summed mean intensities *I*_D_/*I*_1_ of successive data sets (Garman, 2010 ▶) can be used to track the progression of damage in reciprocal space within a certain range, but these do not allow comparisons between experiments. Other directly or indirectly observable experimental proxies, such as the unit-cell size increase and mosaicity increase, do not relate to absorbed dose in a systematic manner (Murray & Garman, 2002 ▶; Ravelli *et al.*, 2002 ▶).

Even when dose values are available, these are usually only reported in the publications accompanying new X-ray structure depositions, and they are not systematically reported within the PDB depositions themselves. They are therefore not generally available for experimenters working with many PDB structures. For this reason it is also unknown how many of the deposited PDB structures were determined from X-ray diffraction experiments exceeding the recommended maximum dose limit of 30 MGy (Owen *et al.*, 2006 ▶), and whether they are compromised by radiation-induced structural damage, and, if so, to what extent. This information, if available, might help those who use PDB structure models derived from X-ray crystallography to avoid treating the deposited models as ‘truth written in stone’ (Pozharski *et al.*, 2013 ▶).

Deposited PDB structure models contain coordinate records for every observed atom in the molecule. For each atom, information relating to the protein’s primary sequence is stored alongside its positional model. The positional model consists of the Cartesian coordinates of the mean atom location within the unit cell, an occupancy value, and one (isotropic) or six (anisotropic) atomic *B*-factors, also called atomic displacement parameters. The decision of whether only one parameter per atom or full anisotropic refinement can be used is mostly informed by the quantity of experimental data available. A model with full anisotropic *B*-factor details has a much larger number of parameters to be refined. The model therefore depends on the availability of a greater number of observations, specifically, a high number of unique reflections in the diffraction pattern, the number of which is itself a function of the resolution of the diffraction. In this study only non-hydrogen atoms and isotropic atomic *B*-factors were considered. The more informative anisotropic atomic *B*-factors were not used in the study reported here, since, due to the large number of degrees of freedom and the associated requirement of high-resolution data, there are relatively few^1^ deposited structures which contain them. The atomic displacement parameters describe the reduction in scattering assumed from a point source due to that source having a range of positions. They can also be understood to describe the uncertainty on the location of the atom (*i.e.* variance in the coordinates), including uncertainty caused by thermal and crystalline disorder. Given a PDB file, these parameters are the only available starting point for an atomic metric describing the specific radiation damage that has been suffered by the structure.

We present an investigation of whether it is possible to find evidence for, and quantify, the extent of specific radiation damage in refined deposited PDB structures using atomic *B*-factors in a new metric, *B*_Damage_. *B*_Damage_ aims to describe the sustained site-specific radiation damage, without resorting to the calculation of structure factors or the inspection of electron density maps. The descriptive value of *B*_Damage_ is established and validated using the dataset of Nanao *et al.* (2005 ▶). This is a set of 12 models, obtained from a low-dose and a high-dose dataset collected from six crystals, each of a different protein.

As mentioned above, when multiple copies of susceptible groups are present within the same protein, they do not damage uniformly at equal rates. The reasons for this ‘preferential’ specific radiation damage are unclear. In the study reported here, *B*_Damage_ is applied to a representative subset of deposited PDB structures in a statistical survey.

Correlations between specific damage and various physicochemical parameters, such as residue types, protein secondary structure, disulfide bond conformations and solvent accessibility, are investigated to provide a basis for theories regarding the causes of preferential specific damage.

## 2. Methods   

Atomic *B*-factors in PDB models usually range from 0 Å^2^ to 80 Å^2^ (see distribution shown in Table S1 of the supporting information^2^), and can, in the absence of translation/libration/screw groups (Schomaker & Trueblood, 1968 ▶; Painter & Merritt, 2006 ▶) or similar approaches, be refined independently. An atomic *B*-factor of 0 Å^2^ represents a non-vibrating atom in an identical position in all unit cells.

Although technically an occupancy value is also present for each atom to indicate the probability of the said atom being observed around the given location, its value is usually fixed to unity. It can be used to encode alternate conformations of residues, but in most cases the experimenter will manually set an ‘even’ occupancy value and will not refine the occupancy (see Table S2 for the distribution of occupancy values in the test PDB set), thus making the occupancy value unsuitable for radiation damage identification purposes.

Atomic *B*-factors are known to correlate with packing density: atoms within more densely packed regions tend to have lower atomic *B*-factors (Weiss, 2007 ▶). Thus atomic *B*-factors cannot be used as a reliable damage metric on their own. By partitioning the protein into distinct packing density volumes, a new atomic locality-independent damage metric, *B*_Damage_, is defined in this work. For this, the concept of packing density must first be considered.

### 2.1. Packing density   

The packing density of an atom or residue can be established by using metrics such as the Ooi number (Nishikawa & Ooi, 1986 ▶), cx ratio (Pintar *et al.*, 2002 ▶) or atomic contact number (ACN; Weiss, 2007 ▶). These respectively describe the number of carbon alpha atoms (Cα) within a given radius of the Cα atom of a residue (Ooi), the ratio between the occupied and unoccupied volume within a given radius (cx), and the number of non-hydrogen atoms within a given radius around each atom (ACN). For all three metrics (Ooi, cx, ACN) the radius can be freely chosen. A small radius up-weights local differences; a larger radius shifts the focus towards global protein properties. Radii between 6 and 8 Å are typically used (Nishikawa & Ooi, 1980 ▶; Halle, 2002 ▶; Weiss, 2007 ▶); Pintar *et al.* (2002 ▶) recommend 10 Å, but radii up to 14–18 Å have also been chosen (Nishikawa & Ooi, 1986 ▶).

If the radius used is too small, then the packing information is strongly influenced by the local residue surroundings, and the possible spread in numerical values for the packing density metric is low. In the work presented here, the packing density metric is only used to provide a partitioning of the protein into volumes of similar packing density. A low spread in packing density values increases the influence of random (stochastic) effects and noise, thus a suitable packing density should have a minimum radius of 8 Å. With an increasing radius, the spread in the numerical values of the atomic contact numbers increases (Figure S1 of the supporting information). However, if the radius is too large, then the effects of protein secondary structure are smoothed over and the packing density information is blurred throughout the macromolecule. Again this means that the partitioning is adversely affected.

When calculating the packing density, account is taken of the neighbouring copies of the protein *via* crystallographic symmetry operators, since radiation damage occurs in the context of a protein crystal, not just on an isolated molecule. Typical packing densities for different secondary structures are shown in Table S3.

### 2.2. *B*_Damage_   

**Figure 1:**
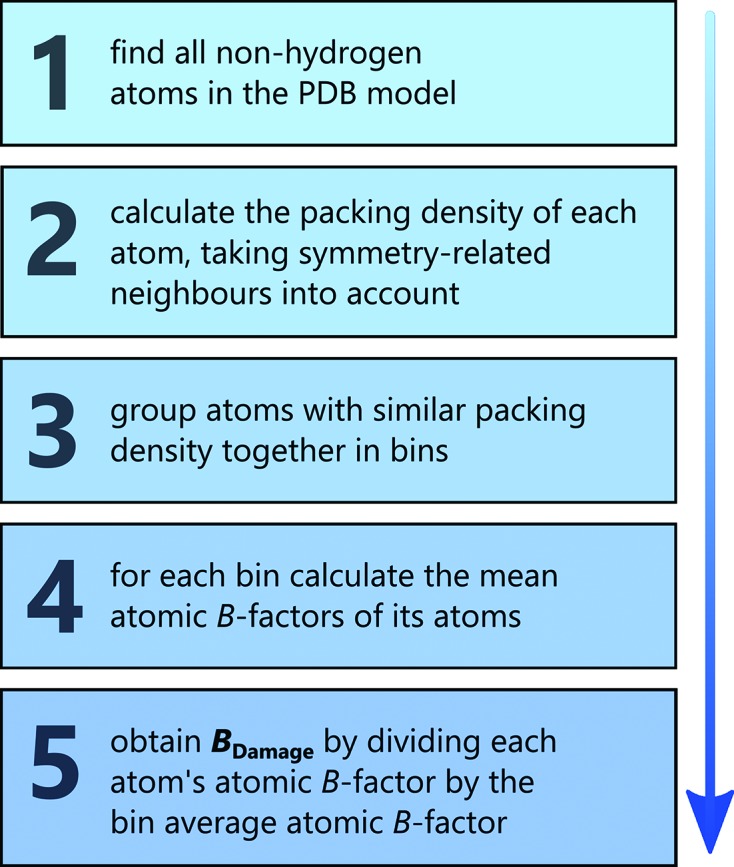
Method of calculating the *B*_Damage_ metric. The full mathematical details can be found in Fig. S2 of the supporting information.

The *B*_Damage_ value of a particular atom *a* is defined as the atomic *B*-factor of that atom, 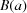, normalized by the average atomic *B*-factor 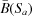 of all non-hydrogen protein atoms *S* within the same PDB structure in a packing density environment similar to that of *a*. An overview is given in Fig. 1 ▶, and the mathematical description can be found in Fig. S2 of the supporting information. *B*_Damage_ is based only on information available in a PDB structure coordinate file.

In this study the packing density environment was defined using atomic contact numbers (ACNs) with a radius of 14 Å. At 14 Å, ACNs regularly lie between 100 and 600 (Fig. S1). For the partitioning of the protein volume, similar packing densities need to be binned together. The exact definition of ‘similarity’ of packing densities is a parameter within *B*_Damage_. If the bins are too small, noise is introduced, whereas, if they are too large, this can cause the loss of information detail. Here, a similar packing density was assumed if, and only if, the division of the ACNs of two atoms by 10 without remainder gave the same result (*i.e.* atoms in the same ACN bin of width 10 were assumed to have similar packing density).

To test the stability of the *B*_Damage_ metric and the reliability of our results, all the tests presented in this study were repeated with ACNs calculated with radii of 8 Å, 13 Å and 13.5 Å, and the tests all led to comparable outcomes.

*B*_Damage_ is a relative metric, in that substructures more disordered than expected in a particular packing density environment will have *B*_Damage_ values greater than 1. Regions with *B*_Damage_ less than 1 may still have been affected by specific radiation damage, but the value of the *B*_Damage_ metric indicates that the electron density disorder as indicated by the atomic *B*-factor is less than expected for that particular packing density environment.

### 2.3. Reference dataset   

The validity of *B*_Damage_ as a suitable metric was investigated by testing it on a set of six pairs of structures obtained from crystals of six different proteins by Nanao *et al.* (2005 ▶) at 100 K (Table 1 ▶). Each of the structures in a pair was determined from two complete diffraction datasets collected from the same crystal, but with the crystal having been subjected to a high-dose ‘burn’ in between the ‘before’ and ‘after’ datasets. Although several other dose series are available in the PDB, this set of structures was chosen since in the original work they were used for an investigation into the feasibility of radiation-damage-induced phasing (RIP; Ravelli *et al.*, 2003 ▶). For five of the six proteins, the specific radiation damage induced by the unattenuated X-ray ‘burn’ caused sufficient movement or dispersion of cysteine disulfide sulfurs between the collection of the ‘before’ and ‘after’ datasets to allow *de novo* phasing by the RIP method. In the sixth case, ribonuclease A, the ‘before’ and ‘after’ structures were obtained by using a previously determined substructure. The structures represented good candidates for testing *B*_Damage_ since they were all processed using the same protocols with the same software and by the same people, which should help to minimize any systematic protein-to-protein variation in noise. Additionally, for all the structures, *B*-factors were refined per atom, a necessary prerequisite for calculating *B*_Damage_.

With the exception of the two insulin datasets, all datasets were collected with exposures that inflicted an absorbed dose lower than that of the X-ray ‘burn’ phase. The ‘before’ and ‘after’ datasets provide a useful low-dose/high-dose comparison for the six model proteins. The low-dose ‘before’ datasets are of course not zero-dose datasets, so it can be expected that these data will give rise to structures that already contain some specific radiation damage. However, as the datasets are sufficiently different to allow RIP, it is still possible to investigate the specific radiation damage development in further detail.

### 2.4. PDB survey and database   

**Figure 2:**
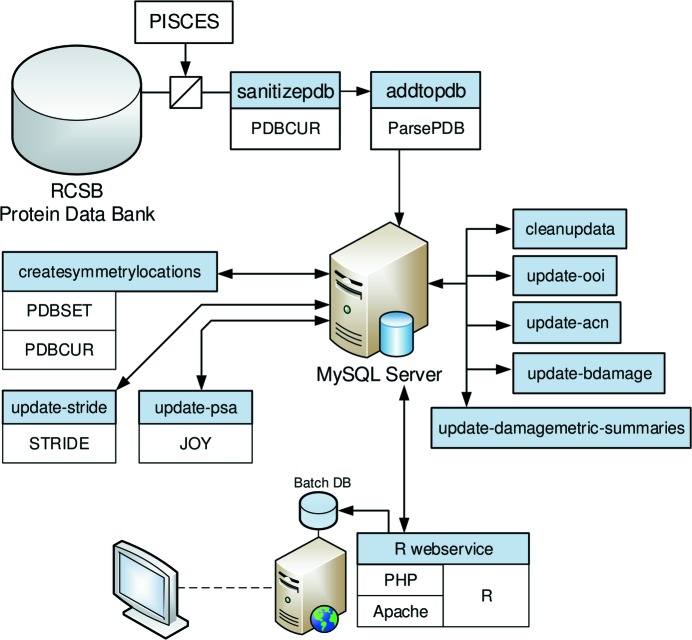
The software and data used to calculate *B*_Damage_ and correlations. The database layout is shown in Fig. S3.

A second larger dataset was developed to consider the systematics of differential specific radiation damage (Fig. 2 ▶). Using the advanced search functions on the PDB website, a set of 11836 PDB protein entries solved by X-ray crystallography at a resolution of between 1.5 Å and 1.8 Å, a refined crystallographic *R*-factor *R*_Work_ 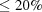 and containing at least one protein chain with a sequence length between 100 and 1000 residues was selected. This limitation to structures of similar resolution is of particular relevance when analysing atomic *B*-factor distributions, as the error on atomic *B*-factors is known to correlate with resolution (Carugo & Argos, 1999 ▶; cited by Weiss, 2007 ▶).

The PDB structure models were downloaded and processed with *PDBCUR* (Winn *et al.*, 2011 ▶) to remove hydrogen atoms, anisotropic *B*-factor information (leaving the separately specified isotropic *B*-factor) and zero-occupancy atoms. For all but 4% of the atoms, the occupancy was 1. If multiple conformations were available, they were replaced by the first conformation with the highest occupancy. The processed PDB files were subsequently parsed (interpreted) by *ParsePDB* (Bulheller & Hirst, 2009 ▶) to extract the chain information, atomic coordinates and atomic *B*-factors. Further PERL scripts were written to extract and calculate ancillary information, such as the locations and dihedral angles of disulfide bonds. All these data were stored in a MySQL database (http://mysql.com) which considerably simplifies the storage and management of large datasets and allows efficient processing and reporting (peak database size: 373 GB; Fig. S3).

Protein structures were placed in their crystallographic context by calculating the position of all symmetry-related atoms. The software *PDBCUR* (Winn *et al.*, 2011 ▶) was used to fill the unit cell with symmetry-related protein copies which were then translated to obtain the atomic coordinates for all non-hydrogen atoms within the 26 spatially neighbouring unit cells. A bounding box around the original protein structure was obtained with *PDBSET* (Winn *et al.*, 2011 ▶) and extended by 14 Å (the chosen radius) in every direction. Symmetry-related atoms that lay within this bounding box were retained and stored in the database. The correctness of the symmetry operations was manually verified using *WinCoot* (Emsley *et al.*, 2010 ▶) and *PyMOL* (http://www.pymol.org) on five PDB entries with different lattice types.

Solvent accessibility information for the molecules was obtained from the software *PSA* (Mizuguchi *et al.*, 1998 ▶), which calculates the relative solvent-accessible area of residues by rolling a water sphere with a radius of 1.4 Å over the van der Waals radii of the atoms.

Secondary structure information was found using *STRIDE* (Frishman & Argos, 1995 ▶). *STRIDE* assigns one of seven secondary structure motifs to each residue, depending on the hydrogen bond energy and backbone torsion angle values.

**Figure 3:**
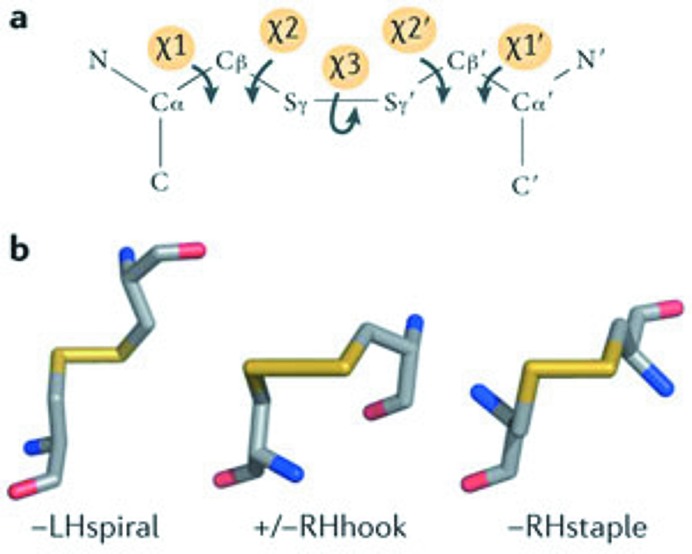
Classification of CYS–CYS disulfide bonds according to dihedral bond angles. (*a*) A disulfide bond can be described by five dihedral angles, which can be defined from either side. (*b*) The three basic groups, spiral, hook, and staple, are determined by the three central angles χ_2_, χ_3_, 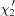 and are named after the motifs they resemble. The sign of the central χ_3_ angle can be used to determine the bond as left-handed (LH) or right-handed (RH), and combined with the signs of the remaining two dihedral bond angles χ_1_ and 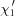 (−, +/−, +) results in a total of 20 different disulfide bond types. Reprinted by permission from Macmillan Publishers Ltd: *Nature Reviews Cancer* (Hogg, 2013 ▶), Copyright (2013).

In many MX studies [see Garman (2010 ▶) for a review] it has been observed that, for metal-free proteins, disulfide bonds are the group most susceptible to damage, so these were the first type of bonds to be investigated. Disulfide bonds were identified in the PDB files and the five dihedral angles that make them up were calculated from the known atomic positions. Using the dihedral angles, disulfide bond conformations can be classified and grouped as spiral, hook, or staple (Fig. 3 ▶). Together with their handedness and the signs of the χ_1_ and 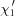 angles, this information can be used to label each disulfide bond as one out of 20 different types (Schmidt *et al.*, 2006 ▶). A preliminary investigation by Hogg & Ravelli (unpublished) suggested that there might be differences in susceptibility to radiation damage between the various disulfide bond groups.

Not every PDB entry can be handled without errors by every program in the processing pipeline described above. To minimize the impact of these errors on the findings reported here, any PDB entry that could not be processed by any relevant software package was removed from the set. The number of structures surviving each step in the pipeline is shown in Table 2 ▶. To ensure that any results were not skewed due to the high multiplicity of certain model proteins in the PDB, a non-redundant list of proteins was selected and retained using PISCES (Wang & Dunbrack, 2003 ▶). Using a sequence identity cutoff of 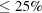, the list of 11707 PDB entries was further reduced to a final non-redundant set of 2704 PDB entries (detailed in Table S4). Of these, 2123 (78.5%) were obtained using diffraction data collected at cryo-temperatures (≤123 K), 61 (2.3%) at room temperature (≥273 K) and 48 (1.8%) at temperatures in-between. The remaining 472 (17.5%) structure models had either no declared experimental temperature or specified clearly unrealistic values. The 61 structures for which the data were recorded at room temperature were omitted from further analysis, leaving 2643 PDB entries.

Two programs were developed to aid in the database analysis and the visualization of results. A PHP script was written to provide an easy-to-use and convenient web service to run jobs written in R script (R Development Core Team, 2011 ▶) containing SQL database queries. These jobs were then picked up by a server application written in R. This simple to set up infrastructure allowed flexible generation of plots and reports, and simultaneously kept a record of all queries and results produced thus far. These programs are available at https://github.com/GarmanGroup/RServer.

To test the validity of the radiation damage metric, it was first tested on the 12 Nanao *et al.* (2005 ▶) datasets. The atomic *B*-factor and *B*_Damage_ distributions for the six low-dose and the six high-dose datasets were compared for all atoms as well as for atoms of residues known to be particularly susceptible to specific damage. The effects of the double X-ray ‘burn’ dose for the ribonuclease A datasets on atomic *B*-factors and *B*_Damage_ values were compared with a normal X-ray ‘burn’ dose for lysozyme. Finally the changes in *B*_Damage_ values between the low- and high-dose dataset were investigated for atoms from specific amino acid residues.

Having established the viability of the *B*_Damage_ metric using these test data, the large representative PDB subset was then analysed.

## 3. Results   

From the results of the analysis described above, evidence for correlations between *B*_Damage_ and physicochemical parameters, such as disulfide bond conformation and solvent accessibility, is presented.

### 3.1. *B*_Damage_ and susceptibility to specific radiation damage   

**Figure 4:**
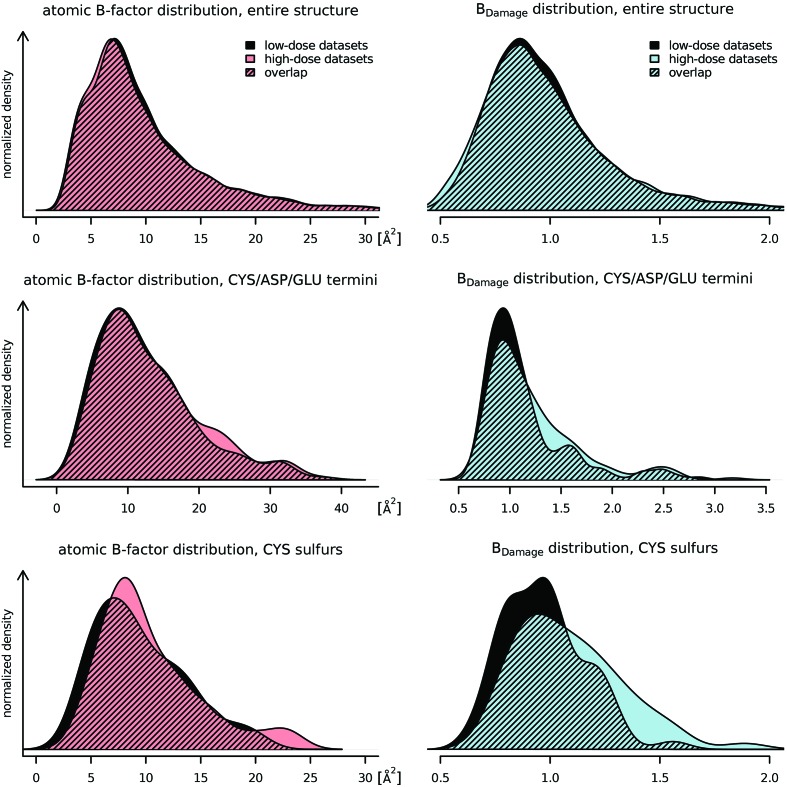
The distributions of atomic *B*-factors (left) and the *B*_Damage_ metric (right) for the low-dose (black) and high-dose (colour) datasets of the six proteins from Nanao *et al.* (2005 ▶) are given. All of the distributions are smoothed using a kernel density estimator (R Development Core Team, 2011 ▶). Top: the distribution for all non-hydrogen atoms (*n* = 7349) shows that both *B*-factor and *B*_Damage_ have uni-modal slightly right-skewed distributions. *B*_Damage_ has a mode below unity and by definition a fixed mean of 1. The differences between the low- and high-dose datasets are minimal: radiation damage causes the atomic *B*-factors to decrease, and the spread of *B*_Damage_ to increase slightly. Middle: only *B*-factors and *B*_Damage_ metrics of CYS Sγ, ASP Oδ and GLU O∊ atoms (*n* = 184) are shown. These atoms are at the end of residue side-chains known to be susceptible to specific radiation damage. Both the atomic *B*-factors and *B*_Damage_ show higher values for the high-dose datasets. The change in *B*_Damage_ is marked and separates specifically susceptible atoms from the bulk of the protein. Bottom: these distributions only show cysteine sulfurs (*n* = 58). The atomic *B*-factors tend to increase, but do not behave consistently. *B*_Damage_ shows a consistent strong shift towards higher values, indicating that the *B*-factor of the sulfur atoms increases much faster than the *B*-factor of other atoms in similar packing densities.

The effects of specific radiation damage on the distribution of *B*-factors and *B*_Damage_ were explored by comparing the low-dose and high-dose Nanao *et al.* (2005 ▶) datasets (Fig. 4 ▶) using Welch two sample *t*-tests. This statistical test is an adaptation of the more familiar Student’s *t*-test, and is used to check the hypothesis that the mean value of two populations are equal but that the two samples possibly have unequal variances. The changes in the distributions for the entire structures, excluding hydrogen atoms, were subtle (Fig. 4 ▶, top): the *B*-factor distribution shifted slightly towards lower values for the high-dose dataset (not significant, *p* = 0.188). The distribution of *B*_Damage_ has, by definition, a fixed mean at 1.0, thus no movement of the mean can be observed (*p* = 1.0). Using Levene’s test (Brown & Forsythe, 1974 ▶), which assesses the equality of variances (homoscedasticity) for a variable calculated for two or more groups, an increase in variance for the high-dose dataset and thus a ‘flattening’ of the distribution can be observed (*p* = 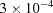).

When considering only the side-chain terminal atoms of amino acid residues known to be susceptible to specific radiation damage (CYS Sγ, ASP Oδ and GLU O∊ atoms; Fig. 4 ▶, middle) a clear difference in the change of the *B*-factor and *B*_Damage_ distributions can be seen. A small increase in the number of atoms with *B*-factors between 20 Å^2^ and 25 Å^2^ was visible, but no shift of the distribution could be observed (*p* = 0.351). However, for *B*_Damage_ there is some evidence for a shift towards higher values (*p* = 0.058), indicating that the increase in *B*-factor for these atoms was larger than that expected from atoms with similar packing densities.

Looking only at cysteine sulfurs, a clearer picture emerged (Fig. 4 ▶, bottom). The atomic *B*-factors tended to increase, yet the overall change in the distribution of *B*-factors was insignificant (*p* = 0.257). The change in *B*_Damage_, however, clearly showed that cysteine sulfurs were affected by the increasing dose (*p* = 0.003).

To investigate the potential of the *B*_Damage_ metric further, difference electron density peak heights for atoms known to be susceptible (cysteine Sγ and the aspartate and glutamate terminal O atoms) were calculated from the before and after models (*F*_before_ − *F*_after_) using *SHELXC* (Sheldrick, 2010 ▶) and *ANODE* (Thorn & Sheldrick, 2011 ▶) and plotted against *B*_Damage_. The scatterplot for all the Sγ atoms from the six test proteins (Fig. S4) shows an indication of a positive correlation (correlation coefficient CC = 0.25, *p* = 0.05), but no significant correlation for the GLU and ASP O atoms (CC = −0.15, *p* = 0.09). This latter result is not too surprising, given the weak nature of the difference density for the terminal O atoms at the doses used in the test datasets of Nanao *et al.* (2005 ▶). However, it would be instructive to know at what level of damage such a correlation might become evident, and this question is currently being further investigated. If *B*_Damage_ is to be a useful metric for looking at individual PDB files to find damaged sites, understanding at what level of radiation damage severity it will identify such atomic sites is clearly of interest.

However, these results together indicate that *B*_Damage_ is a metric sensitive enough to distinguish between the more stable regions of the protein and those regions sustaining specific radiation damage.

### 3.2. *B*_Damage_ and correlation with dose   

**Figure 5:**
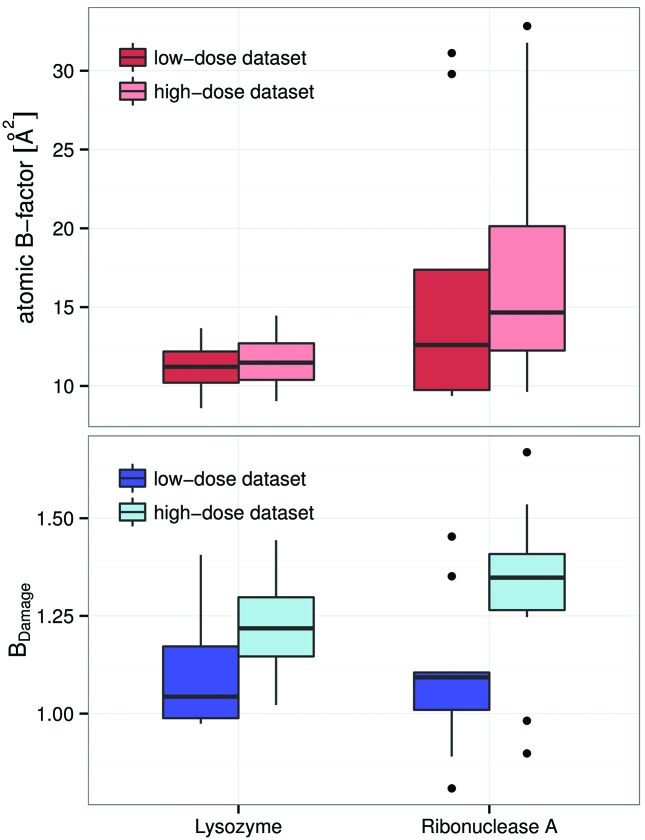
Radiation damage to GLU O atoms. Top: lysozyme (left, four GLU O atoms) was subjected to a 2 MGy X-ray burn, ribonuclease A (right, ten GLU O atoms) to a 4 MGy burn. The increase in *B*-factors is barely visible and within a margin of error for lysozyme. The increase for ribonuclease A is more visible. The coloured box of the boxplot indicates the interquartile range. Bottom: *B*_Damage_ shows a significant change in the GLU O atoms, indicating that this part of the protein degrades more rapidly than other comparable sites. The difference in *B*_Damage_ is larger for ribonuclease A (right), consistent with its exposure to a significantly higher dose.

If *B*_Damage_ is able to identify specific radiation damage it should show larger changes for atoms which have suffered a higher absorbed dose. Here the effects of the X-ray burn on atomic *B*-factors and *B*_Damage_ of the highly susceptible GLU O∊ atoms were compared. In the reference dataset, the ribonuclease A crystal was subjected to an X-ray burn with an effective dose difference between the ‘before’ and ‘after’ datasets of 4 MGy, whereas the other five proteins only suffered an X-ray burn giving a dose of 2 MGy (Table 1 ▶). As can be seen in Fig. 5 ▶, atomic *B*-factors indicate no visible change for GLU O∊ atoms in lysozyme and little change in ribonuclease A. In contrast, the *B*_Damage_ metric achieves a much clearer separation between the low-dose and high-dose datasets, once again suggesting its reliability as a damage metric. Similar results can be observed for the ASP Oδ atoms (Fig. S5).

### 3.3. *B*_Damage_ for different atoms in different proteins   

**Figure 6:**
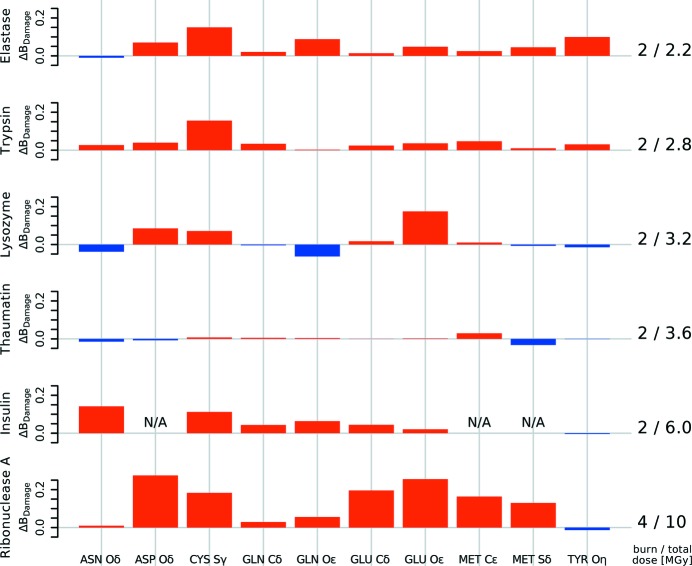
Change in *B*_Damage_ between low- and high-dose datasets for different residue atoms. The change in the median *B*_Damage_ for ten groups of atoms of the six protein structures before and after an X-ray burn is shown. Insulin does not have ASP or MET residues. Different proteins show different *B*_Damage_ patterns. *B*_Damage_ tends to increase for most atoms shown, including ASP Oδ, CYS Sγ, GLU Cδ and O∊, MET C∊ and Sδ. This indicates that these atoms damage faster than others within the same protein, and correlates well with the known specific damage decay pattern of amino acid residues.

The differences in the median *B*_Damage_ for the terminal atoms of known specific radiation-damage-sensitive residues and control residues for the six proteins from Nanao *et al.* (2005 ▶) are shown in Fig. 6 ▶.

Since only ribonuclease A was subjected to a more intense X-ray burn, additional signs of advanced specific radiation damage can be expected. As before, there is a distinct increase in *B*_Damage_ between the low- and high-dose state for the O atoms of the carboxylates in the aspartic acid and glutamic acid in the ribonuclease A model. This indicates that these groups suffered specific damage above the background rate of damage between the low- and high-dose dataset. The asparagine and glutamine residue O atoms show no significant change in *B*_Damage_, which is consistent with the established knowledge that these amino acid residues only become susceptible to specific radiation damage rather later in the process (Juers & Weik, 2011 ▶). *B*_Damage_ also increases in the ribonuclease A model at methionine C∊s, which are cleaved off only at higher doses. The relevant median atomic *B*-factors do not show this significant change for either aspartic or glutamic acid, or for methionine residue atoms (not shown).

The other five proteins in the Nanao *et al.* (2005 ▶) set do not show this marked *B*_Damage_ behaviour at aspartic acid, glutamic acid or methionine residues due to the lower absorbed doses of the ‘burn’.

With the exception of elastase, there is no change in *B*_Damage_ for tyrosine O atoms, indicating that tyrosine residues have not yet suffered detectable damage.

### 3.4. Relationship between *B*, *B*_Damage_ and physicochemical parameters   

*B*_Damage_ has a uni-modal slightly right-skewed distribution with its mode below 1 and a mean value of 1 (Fig. 4 ▶). *B*_Damage_ cannot take negative values, yet its distribution can still be assumed to be approximately normal due to its small standard deviation, thus allowing the use of simple linear regression to analyse the relationship between *B*_Damage_ and physicochemical parameters of selected residues and atoms.

#### 3.4.1. Secondary structure   

**Figure 7:**
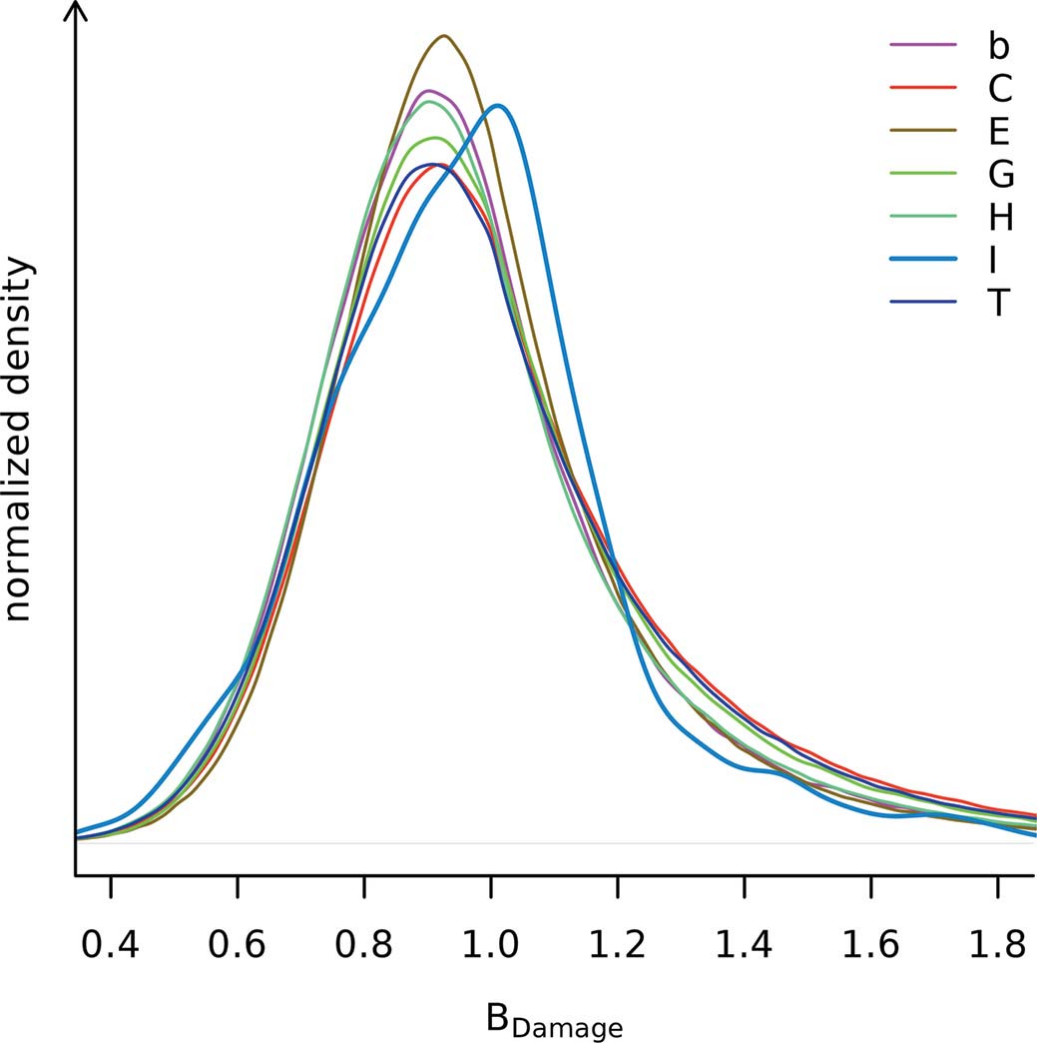
*B*_Damage_ distribution for different protein sections as identified by *STRIDE* secondary structure annotation. The seven secondary structure labels are: alpha helix (H), 3–10 helix (G), π-helix (I), extended conformation (E), isolated bridge (b), turn (T) and coil (C, none of the above) (Frishman & Argos, 1995 ▶). 33.2% of all residues within the PDB subset were marked alpha helix, 23.4% extended conformation, 19.9% turn, 17.9% coil, 4.3% 3–10 helix, 1.2% isolated bridge, and 0.016% π-helix. Applying Levene’s test with median centres (Brown & Forsythe, 1974 ▶) for equal variance on a sample of 1500 atoms from each of the seven secondary structure labels, different variances are confirmed (*p* < 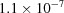). All of the distributions are smoothed using a kernel density estimator (R Development Core Team, 2011 ▶).

Visual inspection of the distribution of *B*_Damage_ values in the PDB subset indicates that, with the exception of π-helix regions, *B*_Damage_ is independent of the secondary structure as determined by *STRIDE* (Fig. 7 ▶). It is possible that the different behaviour of the π-helix region may be caused by sampling effects since these regions are much rarer than any other secondary structure and account for only 0.016% of all residues in the PDB subset.

#### 3.4.2. Disulfide bonds   

**Figure 8:**
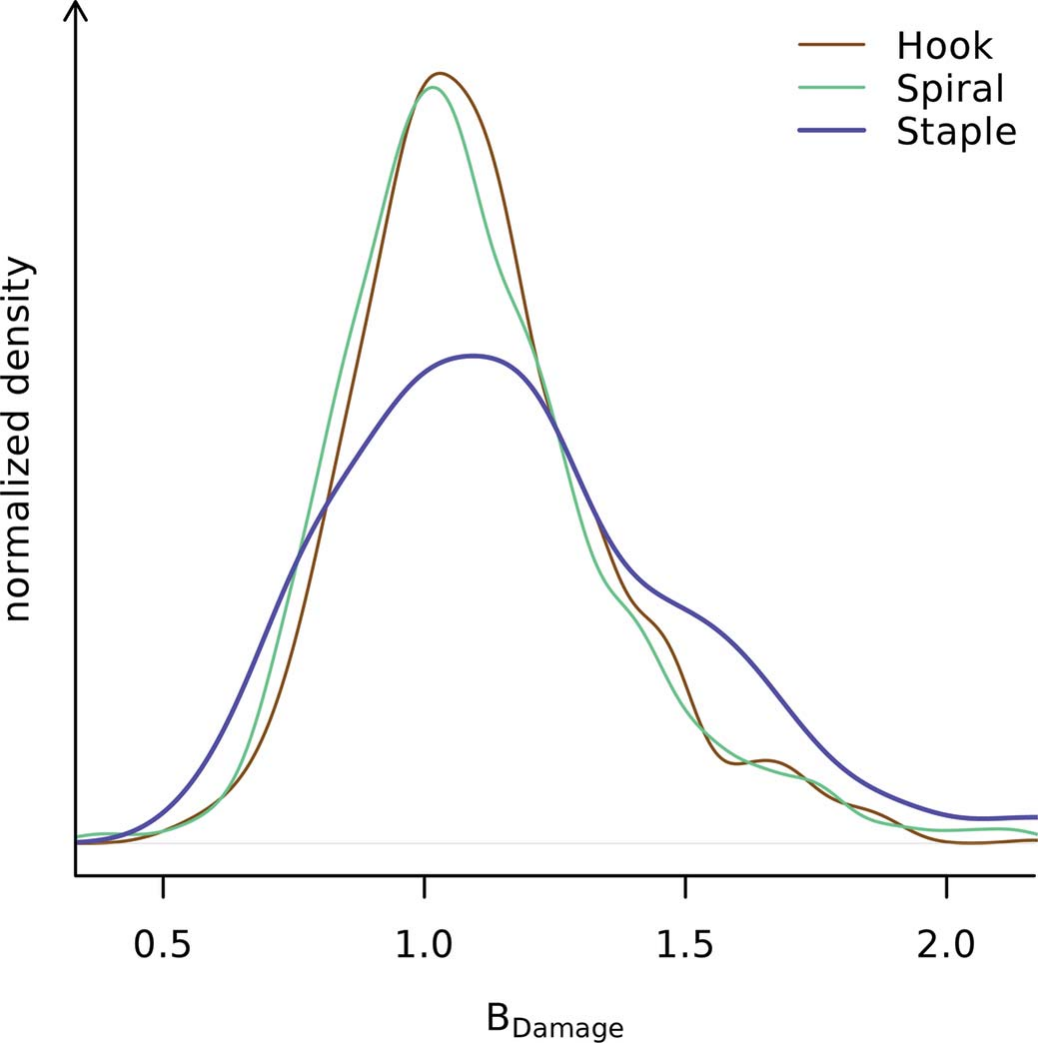
*B*_Damage_ distributions of disulfide sulfurs stratified by disulfide bond group. Of the three disulfide bond groups (hook, *n* = 1070; spiral, *n* = 1424; and staple, *n* = 470) the *B*_Damage_ distribution of staple disulfide bonds deviates from the other two. The three *B*_Damage_ distributions have different variances (*p* < 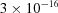), indicating there is an underlying difference between them. The variances of the atomic *B*-factor distributions are similar (*p* > 0.05; not shown). All of the distributions are smoothed using a kernel density estimator (R Development Core Team, 2011 ▶).

The three different basic disulfide bond groups show different *B*_Damage_ distributions in the PDB subset (Fig. 8 ▶). The inequality of variances of the *B*_Damage_ distributions can be confirmed by again using Levene’s test (*p* < 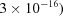. The distributions of the atomic *B*-factors do not show different variances (*p* > 0.05, data not shown) and in addition there is no evidence for them having different mean *B*-factors (*p* > 0.24). While statistical analysis with *ANOVA* is precluded by the different variances, the fact that the *B*_Damage_ distributions for different disulfide bond groups show different variances indicates that there may be a systematic change in the damage behaviour of the three disulfide bond groups. Staple-group disulfide bonds show a tendency towards higher *B*_Damage_ values. Further detailed analysis of the 20 disulfide types within the three broad groups is limited by the large variation in sample sizes (*e.g.* only two disulfide bonds of type +LHStaple are observed, but 790 of type −LHSpiral).

#### 3.4.3. Packing density   

As expected there is strong evidence for a negative correlation between atomic *B*-factors and ACNs, *i.e.* more densely packed regions of the protein contain atoms with a lower *B*-factor (*p* < 0.001; data not shown) for both the low-damage and high-damage reference sets as well as the PDB subset. This matches the observations of Weiss (2007 ▶), who demonstrated that packing density can be used to predict atomic *B*-factors. In contrast, the overall correlation between *B*_Damage_ and the atomic contact numbers of all non-hydrogen atoms is zero. This property directly follows from the definition of *B*_Damage_, since it is a metric normalized by the average *B*-factor of atoms with similar ACNs.

#### 3.4.4. Solvent accessibility   

**Figure 9:**
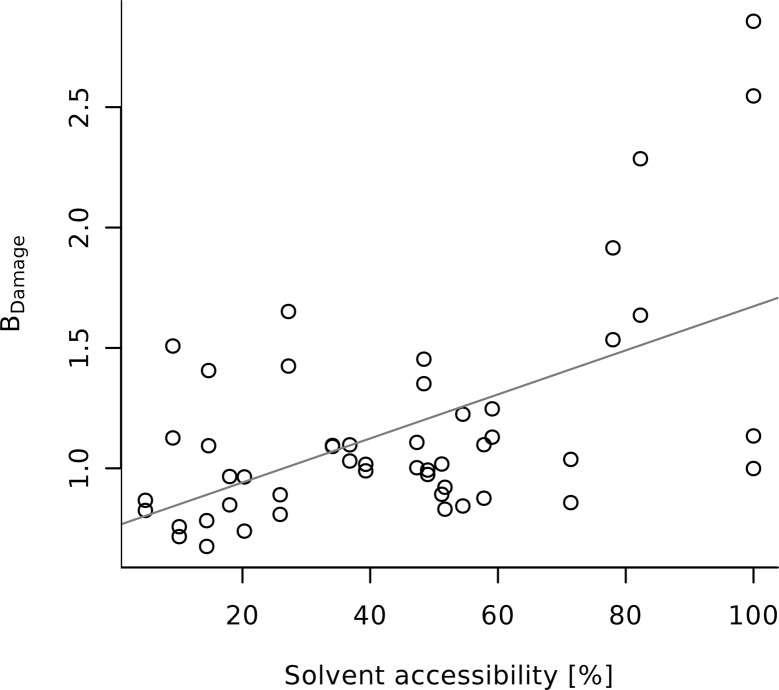
The *B*_Damage_ values of GLU O∊s of the six proteins in the Nanao low-dose dataset are positively correlated to their solvent accessibility. The solvent accessibility is given as a percentage of the residue surface that is accessible to solvent as calculated by the program *PSA* (Mizuguchi *et al.*, 1998 ▶). A list of correlations between the solvent accessibility and other atoms across the datasets are given in Table 3 ▶.

The Nanao *et al.* low- and high-damage reference sets show significant correlation between the per-residue solvent accessibility and *B*_Damage_ for the whole protein as well as for entire ASP, CYS, GLU and TYR residues (*p* < 0.001). There is also some evidence of correlation with the radiation damage prone termini of ASP, CYS, GLU (see Fig. 9 ▶), as well as TYR side-chains and MET residues (see Table 3 ▶: *p* < 0.05). Additionally, the data hint at a strong effect with dose for the MET/C∊, but this is not statistically significant (*p* > 0.1; *n* = 11).

For the PDB subset there is solid evidence for correlations between the residue solvent accessibility and *B*_Damage_ for all ASP, CYS, GLU, MET and TYR residues and their side-chain termini. However, the observed effects are consistently weaker in the PDB subset than in the Nanao *et al.* reference datasets.

## 4. Discussion   

The data presented here show an evaluation of the *B*_Damage_ metric using the six pairs of datasets from Nanao *et al.* (2005 ▶) and an investigation into *B*_Damage_ correlations in a broad sample of deposited PDB structure models.

### 4.1. Nanao dataset   

Atomic *B*-factors contain a strong packing density dependent component, which dominates information useful for assessing specific damage. *B*_Damage_ highlights areas of specific damage, which are not visible from using atomic *B*-factors alone. Specifically damaged residues, such as the glutamic acid residues in lysozyme and ribonuclease A, can be identified. The preferential damage between residues of the same type can be compared using *B*_Damage_ values. The value of the *B*_Damage_ metric was shown by applying it to a set of proteins (Nanao *et al.*, 2005 ▶) and comparing the results with expected and established protein specific damage patterns. The applicability of *B*_Damage_ was demonstrated by investigating the changes in specific damage with increasing dose.

Care must be taken not to generalize the relationship of *B*_Damage_ and dose from the observations on this small set of proteins. While all six of the Nanao *et al.* (2005 ▶) proteins were subjected to different doses, only the ribonuclease A protein crystal was subjected to a more intense X-ray burn. Aside from this being only a single sample, Nanao *et al.* (2005 ▶) indicate that the specific damage pattern of ribonuclease A may be atypical. They found that its disulfide bonds were not the most susceptible sites within the protein, but rather the most susceptible residues were found on the protein surface and play a role in crystal packing. This, however, is consistent with the observation that the exact and detailed decay patterns vary considerably between different proteins.

### 4.2. PDB dataset   

*B*_Damage_ is particularly useful for large-scale statistical investigations, as it does not depend on human intervention or classification of damage levels, for example *via* the observation of electron density decay, and only requires a single protein structure coordinate model. *B*_Damage_ provides additional new insight over atomic *B*-factors for the representative PDB subset: different disulfide bond groups show different *B*_Damage_ distributions, indicating that staple-group disulfide bonds may be preferentially sensitive to specific radiation damage, or may even undergo a dissimilar specific damage process from other disulfide bonds. According to Schmidt *et al.* (2006 ▶) this group of disulfide bonds includes all allosteric (protein function regulating) disulfide bonds, but not the bulk of structural or catalytic ones. The analysis of the representative PDB subset indicates that, with the possible exception of π-helix regions, the protein secondary structure does not significantly affect the distribution of specific damage.

The PDB dataset also provides strong evidence for a positive correlation between *B*_Damage_ and solvent accessibility for all the residues and residue termini known to be particularly susceptible to specific damage. This is consistent with the results of Petrova *et al.* (2010 ▶) as well as Warkentin *et al.* (2012 ▶). These groups based their damage metric on atomic *B*-factors or, the intrinsically linked, refined occupancy values with the atomic *B*-factors held at a fixed value. These results are in contrast to those of Fioravanti *et al.* (2007 ▶) who used peaks in the electron density difference map to identify sites of damage, and Homer *et al.* (2011 ▶) who investigated real space electron density decay in subsequent data sets. This indicates that the difference between methods for defining ‘damage’ could significantly affect the conclusions that are drawn.

From these results we conclude that *B*_Damage_ appears to be a useful indicator of the radiation damage susceptibility of residues. Since *B*_Damage_ can be calculated from a single PDB file it could potentially be used by the crystallographer during and after refinement to identify candidate locations for radiation damage mediated structural changes in the protein.

The automated identification and quantification of specific radiation damage over a number of PDB structures permits the statistical investigation of preferential specific damage, allowing the use of PDB models that previously were not suitable for radiation damage research. Ultimately it may be possible to use a structure-wide *B*_Damage_ derivate as an overall quality indicator for specific damage. It could be used alongside other established tests (Read *et al.*, 2011 ▶) during structure deposition to ensure the accuracy and veracity of published protein structure models.

### 4.3. Limitations   

*B*_Damage_ depends, by its nature, on high-quality PDB structure models. It should only be applied to PDB files containing atomic *B*-factors that were refined per-atom. *B*_Damage_ in its given definition does not allow for the use of multiple models and partial occupancies. Unmodelled regions of the protein are particularly problematic, as they affect both the average atomic *B*-factor calculations and the packing density calculations. *B*_Damage_ does not currently make use of the information available in anisotropic atomic *B*-factors.

It can be postulated that a protein-wide variant of *B*_Damage_, rather than its current description at an atomic level, might be used to indicate the level of structural damage sustained during the diffraction experiment, and could be used as a quality control measure upon PDB structure deposition.

## 5. Summary   

We have presented a metric, *B*_Damage_, that takes a single refined PDB structure model and extracts per-atom information on relative specific damage. *B*_Damage_ highlights areas of the protein where radiation mediated structural changes may have taken place. It could be used by a crystallographer inspecting a single structure for such changes, as well as for statistical investigations to provide insight into the causes of preferential specific radiation damage decay, and may ultimately aid the understanding of specific damage mechanisms.

## Supplementary Material

Tables S1-S4; Figs S1-S5. DOI: 10.1107/S1600577515002131/xh5047sup1.pdf

## Figures and Tables

**Table 1:** The low- and high-dose structures of six protein crystals (Nanao *et al.*, 2005 ▶)

	Elastase	Insulin	Lysozyme	Ribonuclease A	Thaumatin	Trypsin
PDB structure before burn	2blo	2bn3	2blx	2blp	2blr	2blv
Dose per dataset	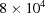 Gy	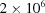 Gy	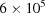 Gy	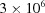 Gy	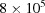 Gy	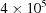 Gy
Dose X-ray burn	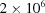 Gy	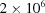 Gy	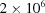 Gy	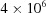 Gy	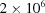 Gy	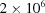 Gy
PDB structure after burn	2blq	2bn1	2bly	2blz	2blu	2blw
Number of CYS/TYR/ASP/GLU/MET residues	8/11/8/4/2	6/4/0/4/0	8/3/7/2/2	8/6/5/5/4	16/8/12/6/1	12/10/6/4/2

**Table 2:** Number of PDB structures in different stages of the selection process

Proteins structures		Steps in the selection process
11836		Initial selection by PDB search
31	(0.3%)	Could not be processed by *PDBCUR*
23	(0.2%)	Could not be processed by *ParsePDB* (PDB file contains multiple models)
6	(0.1%)	Could not be processed by *ParsePDB* (PDB file curation errors)
4	(0.0%)	Curating errors regarding disulfide bond declarations
65	(0.5%)	Could not be processed by *STRIDE*
0	(0.0%)	Could not be processed by *JOY*/*PSA*
11707	(98.9%)	Structures successfully processed
2704		Non-redundant structures selected by *PISCES*
61		Structures resulting from room-temperature diffraction experiments
2643		Structures used in this study

**Table 3:** Slopes of simple linear regression models predicting *B*_Damage_ from solvent accessibility for selected atoms of the Nanao *et al.* (2005 ▶) datasets and the PDB dataset The intercept values are not shown. Significant correlations with *p* 0.05 are marked with an asterisk [linear correlation coefficient, *t*(*n* 2) distribution].

Residue/atoms	Nanao *et al.* (2005 ▶) low-dose datasets	Nanao *et al.* (2005 ▶) high-dose datasets	PDB subset
ARG N	0.901*	0.915*	0.574*
ARG C	0.432	0.458	0.615*
ARG N	0.700*	0.710*	0.643*
ASP O	0.670*	0.687*	0.259*
CYS S	0.550*	0.676*	0.286*
MET S	2.041	3.326	0.438*
MET C	3.266	5.807	0.338*
GLN N	1.445*	1.453*	0.432*
GLN O	0.921*	0.989*	0.393*
GLU O	0.914*	0.933*	0.309*
TRP N	0.898*	1.124*	0.260*
TYR O	0.667*	0.681*	0.305*

## Footnotes

1 Out of 11836 PDB candidate structures, 3206 (= 27%) contain anisotropic atomic *B*-factors. Of the 2704 selected representative structures used in this study, 936 (= 35%) have anisotropic atomic *B*-factors.

2 Supporting information for this paper is available from the IUCr electronic archives (Reference: XH5047).
