# Supplementary material for: Identifying and quantifying radiation damage at the atomic level
Source: J Synchrotron Radiat. 2015 Feb 14;22(Pt 2):201–12. doi: 10.1107/S1600577515002131 (PMC4344357; doi:10.1107/S1600577515002131)
Supplement: Supplementary file 1 [file s-22-00201-sup1.pdf]

JOURNAL OF SYNCHROTRON RADIATION

Volume 22 (2015)

Supporting information for article:

Identifying and quantifying radiation damage at the atomic level

Markus Gerstel, Charlotte M. Deane and Elspeth F. Garman

Identifying and quantifying radiation damage at the atomic level

Gerstel, M., Deane, C. M. & Garman, E. F. (2015).

J. Synchrotron Rad. **22**, doi:10.1107/S1600577515002131.

Table S1 Isotropic atomic *B*-factors present in the PDB subset.

Atomic *B*-factor [Å²]	Number of atoms	Percentage of atoms
$B < 0$	3	<0.01 %
$B = 0$	4,610	0.05 %
$0 < B < 80$	10,143,262	99.65 %
$B \geq 80$	31,338	0.31 %

Atoms with negative atomic *B*-factors were found in PDB structure entry 3cls.

Table S2 Occupancy values present in the PDB subset.

Occupancy *occ*	Number of atoms	Percentage of atoms
$occ < 0$	5	<0.01 %
$occ = 0$	9,177	0.09 %
$0 < occ < 0.5$	68,511	0.67 %
$occ = 0.5$	234,192	2.30 %
$0.5 < occ < 1$	72,020	0.71 %
$occ = 1$	9,795,303	96.23 %
$occ > 1$	5	<0.01 %

Some PDB structures contain occupancy values outside the valid probability range: the structure entries 1ie0 and 1wul report occupancies above 1, and the structure 3hkw reports occupancies below 0.

Table S3 Packing densities of protein secondary structure.

Density metric, radius	Secondary structure	Min	Mean	Max	Variance	Secondary structure	Min	Mean	Max	Variance
Ooi, 8 Å	b	4	11.5	21	6.1	H	4	10.5	22	5.5
	C	1	9.7	22	8.5	I	4	12.4	20	6.9
	E	4	12.0	23	5.1	T	8	9.6	22	8.1
	G	4	9.5	21	7.1					
Ooi, 13 Å	b	10	43.4	72	89.5	H	8	42.9	80	103.6
	C	3	39.7	78	106.2	I	22	48.8	71	136.1
	E	11	47.0	84	93.6	T	5	38.6	82	106.8
	G	8	39.6	75	104.2					
Ooi, 14 Å	b	11	53.1	90	132.1	H	8	52.6	100	148.6
	C	3	48.9	95	153.9	I	29	59.9	82	211.8
	E	12	58.0	102	141.3	T	5	47.8	96	154.3
	G	9	49.1	96	150.4					
ACN, 8 Å	b	13	92.3	141	333.9	H	8	89.2	147	360.8
	C	0	81.1	145	444.8	I	22	98.8	141	337.2
	E	8	96.5	145	294.2	T	8	79.9	144	447.6
	G	8	82.2	141	424.6					
ACN, 13 Å	b	72	340.6	531	5,245.9	H	46	336.2	542	5,757.5
	C	11	313.7	541	6,032.7	I	158	385.7	517	7,705.1
	E	46	366.8	547	5,191.2	T	35	306.7	540	6,229.6
	G	48	314.5	539	6,074.3					
ACN, 13.5 Å	b	74	376.7	589	6,416.0	H	50	372.4	600	6,973.5
	C	14	348.1	597	7,293.6	I	173	428.4	570	9,721.7
	E	53	405.5	601	6,377.9	T	37	340.8	597	7,541.5
	G	51	349.3	602	7,341.6					
ACN, 14 Å	b	80	415.3	654	7,790.0	H	52	410.9	666	8,366.2
	C	16	385.1	658	8,757.2	I	190	474.0	633	12,043.5
	E	56	446.8	668	7,762.1	T	38	377.4	666	9,056.6
	G	57	386.4	659	8,809.8					

Range, mean and variance of different packing density metrics applied to the 2,704 selected PDB structures. Both ACN and Ooi metrics are counts, so minimum and maximum values are integers. Mean and variance are given to 1 dp. The seven secondary structure labels are: alpha helix (H; n=3,323,956), 3-10 helix (G; n=428,747), π-helix (I; n=1,515), extended conformation (E; n=2,351,831), isolated bridge (b; n=120,856), turn (T; n=1,859,115) and coil (C; n=1,679,478) (Frishman & Argos, 1995).

Table S4 List of the 2,704 selected PDB structures

1A3A 1A3C 1A4I 1A7D 1AKY 1AL3 1ATZ 1B2L 1B2P 1B4V 1B4Z 1B5E 1B6A 1B8O 1BQC 1BRT 1BW9 1BX4 1C7S 1CJW 1CQX 1CZF 1D2N 1D8W 1DBW 1DEO 1DGW 1DJ0
1DJE 1DK8 1DLW 1DPS 1DQG 1DQZ 1DWK 1DYP 1DYQ 1E19 1E2R 1E2W 1E30 1E42 1E4C 1E5M 1E6Y 1E71 1E7R 1EDG 1EEX 1EJD 1ELK 1ELW 1EOK 1EQM 1EWF 1EYE
1EYH 1EZW 1F0L 1F46 1F5V 1F60 1F74 1F7L 1FCQ 1FJ2 1FJJ 1FN9 1FNL 1FO9 1FQT 1FT5 1FTR 1FXO 1G2Q 1G58 1G60 1G6S 1G8M 1GD0 1GGZ 1GKB 1GKK 1GNY
1GP6 1GTJ 1GTK 1GU7 1GUQ 1GV2 1GVG 1GXN 1GXY 1GZJ 1H03 1H14 1H16 1H2B 1H41 1H4C 1H4R 1H4Y 1H6L 1H72 1H9M 1HBZ 1HD5 1HFE 1HH8 1HT6 1HTW 1HW1
1HX6 1HXI 1I0S 1I1N 1I2H 1I3K 1I86 1IAB 1ID0 1IE0 1IIB 1IJB 1IJT 1ILK 1INL 1IOM 1IQQ 1IV2 1J1M 1J1Y 1J27 1J2W 1J7G 1J83 1J8B 1J8U 1J97 1JA9
1JB3 1JG9 1JH6 1JNE 1JNR 1JP4 1JQ5 1JQC 1JSR 1JWQ 1JY1 1JYH 1K1E 1K20 1K4N 1K4V 1K6X 1K75 1K77 1K92 1K9Z 1KAP 1KDG 1KG6 1KGS 1KI0 1KIC 1KLL
1KM4 1KOE 1KP4 1KPT 1KSC 1KTG 1KUI 1L0G 1L2H 1L6P 1L7A 1L9X 1LC8 1LJ8 1LK9 1LMI 1LN4 1LO7 1LOV 1LQV 1LV7 1M0W 1M2A 1M2X 1M4I 1M5T 1M65 1M6K
1M6S 1M7J 1M8U 1MG4 1MIX 1MPG 1MR7 1MSK 1MTY 1MXI 1N45 1N57 1N5Q 1N5W 1N7H 1N8U 1N93 1N9E 1NBC 1NBU 1NC5 1NEP 1NF9 1NH8 1NKG 1NLN 1NN5 1NNH
1NOG 1NPY 1NQD 1NR0 1NS5 1NSZ 1NTH 1NTY 1NU0 1NU3 1NVK 1NWA 1NXC 1NYT 1NZJ 1O1Y 1O1Z 1O3Y 1O4R 1O6V 1O9G 1OA1 1OA4 1OAD 1OC6 1OCY 1OD6 1OF8
1OFL 1OFN 1OFZ 1OGO 1OGQ 1OI2 1OJK 1ONW 1OOE 1OQ1 1OQ5 1ORR 1ORU 1OSY 1OW4 1OWL 1OXS 1OYG 1OYW 1OZ2 1P0Z 1P1J 1P3C 1P3D 1P5D 1P5Z 1P7S 1P90
1P9H 1PB1 1PBY 1PDO 1PG4 1PKK 1PM4 1PMJ 1PMY 1POA 1PU6 1PV5 1PVM 1PX5 1PXV 1PZ3 1Q0G 1Q11 1Q1A 1Q1F 1Q4U 1Q5Z 1Q7E 1Q8D 1Q9U 1QAZ 1QCZ 1QD1
1QF8 1QG8 1QGI 1QH8 1QJP 1QMG 1QNS 1QQ9 1QQJ 1QSA 1QTO 1QTP 1QVY 1QWD 1QWG 1QWO 1QWR 1QZ0 1R0U 1R2R 1R3S 1R4P 1R5L 1R5M 1R7A 1R8M 1R9L 1R9Y
1RCV 1RKI 1RLI 1ROC 1RU4 1RV9 1RW7 1RWG 1RWR 1RXQ 1S0I 1S1D 1S1Z 1S5A 1S99 1S9R 1SBX 1SDI 1SFX 1SG6 1SH8 1SHU 1SK2 1SNG 1ST9 1SYY 1T0B 1T0T
1T1U 1T2W 1T3I 1T61 1T6C 1T6E 1T92 1TAG 1TCA 1TE2 1THG 1TJ6 1TJP 1TLU 1TOA 1TP6 1TP9 1TR0 1TU7 1TWD 1TXG 1TY9 1TZW 1U0K 1U14 1U5D 1U60 1U69
1U6K 1U6R 1U8V 1UAQ 1UAS 1UEK 1UFO 1UGX 1UH4 1UI0 1UJ2 1UKK 1UO9 1UPG 1URR 1US5 1USG 1UT1 1UUQ 1UUX 1UV7 1UX7 1UXL 1UXO 1UZA 1V0T 1V2B 1V3H
1V5D 1V5V 1V7W 1V9F 1VCL 1VDK 1VHF 1VHN 1VIA 1VJF 1VJV 1VK5 1VKA 1VKE 1VKF 1VKN 1VL1 1VL7 1VLA 1VLJ 1VM0 1VMB 1VMJ 1VPB 1VPD 1VPR 1VRM 1VSR
1VYB 1VYI 1W0H 1W1D 1W2W 1W2Y 1W3E 1W4S 1W4W 1W5P 1W6G 1W9I 1WAB 1WBH 1WCU 1WDV 1WEH 1WHI 1WKU 1WLG 1WNY 1WO8 1WOL 1WS8 1WTA 1WTJ 1WUL 1WV3
1WWI 1WWR 1WX4 1WXI 1WZZ 1X1N 1X38 1X3K 1X6O 1X6Q 1X74 1X7D 1X7Y 1X82 1X91 1X9H 1XB3 1XBY 1XC1 1XCR 1XFF 1XFI 1XKP 1XOC 1XOV 1XQL 1XQP 1XSV
1XTE 1XTT 1XV5 1XY7 1Y07 1Y0B 1Y0H 1Y0K 1Y0P 1Y21 1Y2T 1Y7B 1Y7R 1YAC 1YB6 1YBI 1YGT 1YJ7 1YKI 1YLE 1YLL 1YN9 1YO3 1YOC 1YQH 1YRK 1YRW 1YS2
1YSQ 1YT3 1YTL 1YU0 1YUM 1YV1 1YWF 1YX1 1Z0B 1Z3E 1Z3X 1Z6N 1Z7A 1Z9N 1ZBF 1ZD7 1ZHS 1ZI9 1ZJC 1ZLD 1ZMT 1ZRS 1ZVT 1ZW6 1ZXU 1ZY7 1ZZ1 1ZZW
2A0B 2A14 2A2K 2A32 2A35 2A3M 2A4D 2A4O 2A5L 2A65 2A6B 2A6V 2A7B 2A94 2A9D 2A9I 2A9S 2AAL 2ABW 2AD6 2AEX 2AFW 2AG4 2AH5 2AHF 2AII 2AJ6 2AJ7
2AL1 2AML 2APJ 2AR1 2ARC 2ASF 2ATF 2AUO 2AVD 2AXC 2AXQ 2B0P 2B0V 2B2H 2B3L 2B4V 2B5H 2B5W 2B61 2B65 2B7U 2B8M 2BBA 2BDR 2BEK 2BEM 2BHY 2BJF
2BJN 2BJQ 2BJV 2BKM 2BKV 2BMW 2BNM 2BP6 2BRJ 2BSY 2BT6 2BUE 2BV9 2BWR 2BZ1 2C0H 2C2I 2C2P 2C2T 2C54 2C61 2C6Q 2C6U 2C79 2C8B 2C92 2C9Q 2CBO
2CBZ 2CCM 2CD7 2CDO 2CF7 2CFE 2CHC 2CI3 2CIH 2CIS 2CIU 2CJ2 2CJ4 2CJS 2CKI 2CMW 2CO3 2CWL 2CWR 2CX1 2CXA 2CXH 2CXY 2CYJ 2CZ6 2CZL 2D1G 2D1R
2D29 2D4P 2D5B 2D81 2DBY 2DC3 2DE3 2DEJ 2DF8 2DFY 2DG1 2DG5 2DQA 2DRI 2DSK 2DTC 2DTJ 2DUR 2DVM 2DVN 2DVT 2DWU 2DXA 2DY1 2E0P 2E11 2E1N 2E2O
2E3R 2E7A 2E83 2E8G 2EAE 2EB6 2EB9 2EFF 2EG6 2EHG 2EI1 2EIE 2EJ0 2EJN 2ELC 2ELF 2EPN 2ERB 2ET1 2ETJ 2ETV 2EV1 2EVR 2EWR 2EX0 2EX4 2EZ9 2F0C
2F1F 2F1K 2F1N 2F24 2F2B 2F4Q 2F57 2F5X 2F62 2F6D 2F6R 2F7V 2F8A 2F9F 2F9H 2FA1 2FC3 2FCT 2FDV 2FEX 2FGR 2FH1 2FHP 2FI9 2FLU 2FNU 2FOM 2FOZ
2FP1 2FQX 2FR2 2FSR 2FSU 2FSX 2FTN 2FUL 2FUR 2FW0 2FY7 2FYF 2FYG 2FZB 2G0W 2G1U 2G2C 2G40 2G50 2G62 2G82 2G8S 2GA8 2GAI 2GEY 2GFF 2GHS 2GI3
2GIB 2GIY 2GJ4 2GKJ 2GMQ 2GNP 2GS5 2GS8 2GU3 2GUI 2GUY 2GVK 2GWG 2GWM 2GX5 2GZ4 2H1C 2H1T 2H2R 2H6F 2H7J 2H7M 2H8G 2H98 2HA8 2HE2 2HEG 2HHC
2HHJ 2HHP 2HJE 2HKV 2HLY 2HMJ 2HNG 2HP0 2HPL 2HPS 2HQS 2HQY 2HSJ 2HUH 2HX0 2HX5 2HXW 2HY5 2HYT 2HZK 2I3D 2I3H 2I48 2I53 2I5I 2I5R 2I6H 2I74
2I7A 2I7G 2I8D 2I8E 2I9W 2IA1 2IBA 2IBD 2ICA 2ICG 2ICU 2IDL 2IEQ 2IF6 2IG7 2IGI 2IGP 2IIH 2IJA 2IK9 2IKB 2IKK 2IM9 2IMD 2IMH 2IMJ 2IML 2IMZ
2INU 2ION 2IP1 2IPR 2ISB 2IT9 2ITE 2IU5 2IUQ 2IW1 2IWR 2IXD 2IYA 2IZ6 2J0A 2J0P 2J12 2J1S 2J2J 2J43 2J5S 2J6A 2J6G 2J8K 2J97 2J9B 2J9E 2J9N
2J9O 2JB7 2JC5 2JC9 2JCB 2JCN 2JDC 2JE3 2JE8 2JFG 2JG0 2JHM 2JIC 2JK9 2JLP 2NLI 2NML 2NNU 2NOO 2NPT 2NRK 2NRT 2NT0 2NTP 2NUO 2NVF 2NW0 2NW8
2NXF 2NXW 2NYH 2NYI 2O02 2O0M 2O1Q 2O2I 2O2P 2O2X 2O3F 2O3S 2O5V 2O62 2O6P 2O6Y 2O70 2O7I 2O8Q 2OA9 2OB5 2OC3 2OC5 2OD4 2OD5 2OEB 2OFK 2OIT
2OKF 2OKM 2OKO 2OLR 2OMK 2ONS 2OO1 2OOC 2OOK 2OPL 2OQG 2OQZ 2OR7 2ORW 2OS1 2OSA 2OSV 2OT4 2OU5 2OU6 2OUI 2OX6 2OX7 2OY2 2OY7 2OYA 2OYK 2OYO
2OZJ 2OZV 2P0S 2P12 2P14 2P17 2P1F 2P1M 2P3H 2P3P 2P58 2P6W 2P97 2PA7 2PBD 2PBK 2PET 2PFZ 2PKF 2PLR 2PN0 2POR 2PQV 2PRX 2PS1 2PU3 2PWY 2PXR
2PYQ 2PYX 2Q03 2Q0I 2Q0S 2Q35 2Q62 2Q7D 2Q8K 2Q99 2QA1 2QAC 2QAP 2QB2 2QB7 2QEE 2QEU 2QF9 2QG6 2QGD 2QGI 2QGU 2QGY 2QHF 2QHP 2QHQ 2QHS 2QIB
2QIS 2QK1 2QKP 2QL8 2QLW 2QML 2QNI 2QNK 2QNL 2QRU 2QSA 2QSI 2QSW 2QUB 2QUD 2QUO 2QVU 2QW5 2QWU 2QXF 2QZC 2QZT 2QZU 2R1I 2R2C 2R4I 2R4Q 2R78
2R7G 2R85 2R8Q 2R9F 2RBD 2RBG 2RBW 2RCI 2RDC 2RDG 2RDS 2RFG 2RFQ 2RH3 2RIQ 2RJ2 2RKQ 2RKV 2RLD 2SAK 2SGA 2UUR 2UVJ 2UVK 2UVP 2UWA 2UY2 2UYK
2UYQ 2UYT 2UZ1 2V0H 2V2B 2V2G 2V38 2V3Z 2V4V 2V4X 2V5J 2V6A 2V6U 2V75 2V76 2V7K 2VAC 2VAP 2VB9 2VBP 2VBU 2VCL 2VFK 2VFO 2VFT 2VG0 2VG8 2VK6
2VLQ 2VLZ 2VMH 2VOW 2VPN 2VQ2 2VQP 2VRS 2VSM 2VUW 2VV6 2VVE 2VVP 2VXZ 2VY7 2VYW 2W0B 2W0I 2W31 2W3E 2W3P 2W3X 2W52 2W5T 2W61 2W6K 2W7Z 2W86
2W87 2W92 2W9Y 2WAN 2WAO 2WAW 2WBX 2WCM 2WCR 2WCW 2WDC 2WFO 2WFP 2WH6 2WHE 2WHM 2WI8 2WJ9 2WJR 2WKK 2WMF 2WN3 2WN9 2WNB 2WOP 2WOY 2WQK 2WR8
2WTE 2WTG 2WTP 2WU9 2WUQ 2WVF 2WW5 2WXU 2WY8 2WYA 2WYK 2WZ8 2WZC 2WZO 2X1D 2X21 2X2S 2X32 2X3G 2X3N 2X49 2X4J 2X4L 2X4W 2X5C 2X5F 2X85 2X8R
2X8S 2X9O 2X9X 2XBG 2XBU 2XC3 2XDP 2XE4 2XF3 2XFN 2XGR 2XHG 2XJ4 2XLG 2XMI 2XMX 2XOV 2XPP 2XRH 2XRY 2XSU 2XTP 2XU9 2XVS 2XVY 2XWS 2XXF 2XXN
2XXP 2XXZ 2XZ9 2XZE 2XZK 2Y09 2Y0G 2Y1E 2Y1Q 2Y27 2Y2M 2Y2X 2Y3B 2Y3Q 2Y44 2Y4R 2Y51 2Y6X 2Y71 2Y7D 2Y7O 2Y8G 2Y8N 2YAV 2YB6 2YC5 2YET 2YFD
2YFU 2YG2 2YH6 2YHC 2YJG 2YL6 2YMM 2YMV 2YN0 2YNT 2YOA 2YOG 2YR4 2YV9 2YVT 2YWI 2YWV 2YXM 2YXN 2YYK 2Z0J 2Z0T 2Z0X 2Z14 2Z1E 2Z2N 2Z3H 2Z6O
2Z6R 2Z84 2Z8F 2Z9B 2Z9W 2ZA4 2ZAC 2ZAD 2ZB4 2ZBL 2ZBT 2ZCA 2ZCO 2ZDP 2ZFG 2ZFI 2ZFY 2ZGW 2ZHI 2ZIN 2ZKM 2ZM9 2ZQ0 2ZQO 2ZUI 2ZVY 2ZWA 2ZYC
2ZYT 2ZZJ 2ZZX 3A0Z 3A2Z 3A3D 3A47 3A54 3A57 3A77 3A9I 3A9S 3A9Z 3AA0 3AAM 3AB8 3ACG 3AHC 3AHN 3AI9 3AIN 3AJ3 3AJR 3AKC 3ALL 3AML 3AOW 3AQ2
3ARQ 3ASQ 3ATS 3ATV 3AV3 3AWM 3AXD 3B2X 3B49 3B53 3B5E 3B6E 3B7C 3B7I 3B9T 3BA3 3BB0 3BB7 3BB9 3BCW 3BCY 3BEX 3BFM 3BFP 3BGY 3BHD 3BI1 3BI7
3BIQ 3BIY 3BJN 3BL9 3BLZ 3BN6 3BND 3BNG 3BOD 3BOF 3BOH 3BPK 3BPT 3BPZ 3BQC 3BRC 3BS4 3BS6 3BVF 3BWV 3BXP 3BY4 3BY9 3BYQ 3BZT 3C1Q 3C4B 3C5K
3C5N 3C7X 3C8C 3C8E 3C8W 3C9A 3C9Q 3CA1 3CA8 3CAN 3CCG 3CE7 3CEC 3CH4 3CHJ 3CHM 3CI6 3CIN 3CJ1 3CJM 3CJY 3CKC 3CKJ 3CKK 3CL5 3CLA 3CLS 3CNM
3COV 3CPG 3CQL 3CRY 3CT1 3CTZ 3CUI 3CV1 3CVO 3CWR 3CXK 3CXM 3CXN 3CYP 3CZ1 3CZX 3D0F 3D0J 3D33 3D34 3D40 3D59 3D7I 3D8T 3D97 3D9N 3DAN 3DCZ
3DD7 3DDA 3DDO 3DE8 3DEO 3DF8 3DFF 3DFG 3DG9 3DGT 3DHP 3DI4 3DJ8 3DJE 3DKR 3DLU 3DMN 3DN7 3DNU 3DRJ 3DS8 3DT2 3DWG 3DXT 3DXY 3DZA 3E0I 3E0Z
3E11 3E2V 3E3U 3E4W 3E7H 3E9K 3E9T 3EBV 3EC4 3EC9 3ECD 3EDF 3EDN 3EEA 3EF2 3EF3 3EF8 3EFY 3EH1 3EHG 3EJF 3EJN 3EK3 3EKI 3EKL 3ELG 3EMI 3EN0
3EO7 3EOI 3EQN 3EQX 3ER7 3ERJ 3ERP 3ES4 3ESL 3EU3 3EVO 3EW1 3EW8 3EWN 3EZI 3EZM 3F0H 3F0M 3F0P 3F2E 3F43 3F44 3F47 3F4M 3F4S 3F52 3F5R 3F67
3F6J 3F7Q 3F8X 3F95 3F9M 3F9S 3FBU 3FCX 3FD3 3FDH 3FDJ 3FDX 3FF1 3FFR 3FG8 3FGE 3FGR 3FH1 3FIQ 3FJ1 3FKA 3FKC 3FL2 3FLA 3FM2 3FMC 3FN5 3FO8
3FOJ 3FOT 3FP5 3FPF 3FPK 3FPW 3FRQ 3FRR 3FSU 3FT1 3FUN 3FUW 3FVS 3FW2 3FW8 3FWY 3FWZ 3FYB 3G02 3G0M 3G0T 3G3H 3G48 3G7U 3G7X 3G89 3GAE 3GAX
3GBY 3GD0 3GF3 3GF6 3GFP 3GG7 3GK6 3GKJ 3GKM 3GKR 3GMF 3GMG 3GMV 3GN6 3GNL 3GO9 3GOC 3GOQ 3GRS 3GS9 3GT3 3GVO 3GWI 3GWN 3GY9 3GYK 3GZA 3H05
3H09 3H0U 3H2S 3H3L 3H46 3H4O 3H4W 3H4Y 3H51 3H5L 3H68 3H6J 3H6Q 3H7C 3H7H 3H7I 3H7V 3H8G 3H8T 3H93 3H9B 3H9M 3HA2 3HAP 3HBM 3HD4 3HDO 3HDX
3HID 3HJ4 3HJB 3HJR 3HKW 3HL0 3HLZ 3HMZ 3HN0 3HN5 3HNA 3HNH 3HO6 3HOI 3HP7 3HQ1 3HQM 3HQX 3HRP 3HSY 3HTN 3HU5 3HV2 3HVV 3HYQ 3HZ6 3I07 3I0Y
3I0Z 3I1A 3I24 3I47 3I48 3I4Z 3I6I 3I96 3IAR 3IBZ 3IDU 3IEE 3IGS 3IHV 3IJM 3IKB 3ILF 3ILR 3ILS 3ILW 3IM0 3IM1 3IOX 3IQ1 3IRB 3IRP 3IRS 3ISQ
3IT3 3IU5 3IU6 3IUO 3IV4 3IVZ 3JRV 3JS8 3JSC 3JSY 3JU0 3JXS 3JYZ 3JZ9 3K00 3K0B 3K0X 3K1U 3K1W 3K1Z 3K26 3K3C 3K40 3K4I 3K6M 3K6Q 3K7G 3K8W
3KB9 3KBG 3KBY 3KDW 3KEO 3KG0 3KG9 3KGW 3KGY 3KI6 3KIZ 3KL0 3KMH 3KMT 3KMV 3KOR 3KQR 3KS6 3KSN 3KSX 3KT7 3KU3 3KV1 3KVH 3KVS 3KWS 3L00 3L1F
3L1W 3L34 3L3X 3L40 3L4A 3L4E 3L4H 3L4N 3L51 3L6B 3L8Q 3LAT 3LB4 3LCC 3LD7 3LEW 3LFJ 3LFR 3LFU 3LGB 3LGI 3LHE 3LHO 3LI3 3LID 3LIM 3LIO 3LKM
3LLO 3LLP 3LLX 3LM2 3LOG 3LOP 3LRT 3LSC 3LTJ 3LVU 3LX0 3LX3 3LXS 3LY1 3LYG 3LYH 3LZA 3LZN 3LZW 3M0H 3M0J 3M1H 3M1T 3M1U 3M66 3M6L 3M76 3M7O
3M84 3M86 3M9Z 3MAK 3MD9 3MDM 3MDQ 3ME7 3MF7 3MHZ 3MIL 3MK1 3MKO 3ML1 3MMG 3MOK 3MPC 3MT0 3MVU 3MW8 3MWL 3MWZ 3MXN 3MXO 3MXU 3MXZ 3MYV 3MZ2
3MZF 3MZQ 3N03 3N0U 3N0X 3N1E 3N34 3N37 3N6Y 3N79 3N80 3N8I 3N9C 3N9K 3NA5 3NAD 3ND1 3NDD 3NEC 3NEH 3NEU 3NFT 3NFW 3NGV 3NJC 3NJK 3NKQ 3NM6
3NMW 3NNB 3NOH 3NOK 3NPD 3NPF 3NPK 3NR5 3NRE 3NRF 3NRH 3NRS 3NRW 3NRX 3NSL 3NSW 3NSX 3NT1 3NT3 3NUQ 3NV1 3NWE 3NYH 3NZE 3O0Q 3O0Y 3O12 3O2G
3O2H 3O48 3O4Q 3OAM 3OBE 3OBU 3OC9 3OCC 3OCV 3OEB 3OEP 3OF5 3OGH 3OHG 3OIO 3OJC 3OKX 3OLL 3OM1 3OMC 3OMD 3ON1 3ON9 3ONH 3ONO 3OO8 3OOO 3OOS
3OOU 3OQI 3OS4 3OS7 3OSE 3OST 3OSX 3OT9 3OTI 3OTM 3OU2 3OUG 3OUI 3OUL 3OXH 3P02 3P1G 3P2C 3P2E 3P2T 3P3G 3P3V 3P4G 3P97 3P9A 3P9V 3PA6 3PBT
3PCX 3PCZ 3PDD 3PE7 3PFE 3PFT 3PG6 3PGU 3PI6 3PI7 3PJ0 3PJP 3PJY 3PLN 3PM2 3PMD 3PME 3PMS 3PNA 3PO8 3POF 3POH 3POP 3POW 3PPM 3PR6 3PU9 3PUI
3PVH 3PWK 3PYW 3Q0W 3Q18 3Q1C 3Q1F 3Q1N 3Q1P 3Q1X 3Q20 3Q26 3Q2B 3Q3M 3Q49 3Q4R 3Q60 3Q64 3Q6B 3Q7C 3Q7M 3Q7W 3QB8 3QC7 3QF2 3QH6 3QH8 3QHO
3QNM 3QOR 3QOU 3QP8 3QS2 3QSJ 3QSQ 3QUF 3QUT 3QUV 3QWP 3QY0 3QY1 3QY3 3QY9 3QZ4 3R1M 3R26 3R4V 3R4Z 3R5G 3R5S 3R5Z 3R62 3R6H 3R6U 3R8J 3R9Z
3RAY 3RC4 3RD5 3RF0 3RF3 3RGA 3RHG 3RJT 3RJU 3RJV 3RKC 3RLH 3RLK 3RLO 3RLS 3RNL 3RO0 3RPD 3RPP 3RPW 3RPZ 3RQ7 3RQT 3RR6 3RRI 3RT2 3RU6 3RUO
3RUR 3RVC 3RWV 3RZV 3S0E 3S19 3S21 3S3U 3S5B 3S5F 3S5V 3S7P 3S82 3S8M 3S9J 3S9Z 3SAL 3SBF 3SBQ 3SC7 3SCY 3SGH 3SGV 3SH4 3SHG 3SIH 3SJ5 3SK7
3SK9 3SLR 3SM1 3SMJ 3SNS 3SQR 3SS7 3SSX 3ST1 3STD 3SXM 3SZ3 3T01 3T0L 3T0W 3T1K 3T4H 3T4L 3T50 3T63 3T68 3T7A 3T7D 3T7H 3T8G 3T8J 3T8K 3T90
3T92 3T95 3T9G 3T9L 3T9W 3TA5 3TAW 3TBD 3TC2 3TC3 3TC7 3TCQ 3TCV 3TDW 3TE8 3TEF 3TFJ 3TG7 3TGH 3THD 3TIP 3TJ8 3TJL 3TK0 3TKF 3TL1 3TLZ 3TMB
3TNT 3TNY 3TOS 3TQE 3TQL 3TT9 3TUO 3TUT 3TVK 3TW1 3TYT 3U15 3U1D 3U1L 3U26 3U2A 3U2V 3U3L 3U3Z 3U49 3U4V 3U9J 3UB1 3UC1 3UDF 3UF6 3UF8 3UFA
3UFB 3UFE 3UID 3UJD 3UMO 3UOA 3UP0 3UPL 3UPS 3UQ8 3URK 3USH 3UTK 3UUW 3UV9 3UW1 3UW3 3UWS 3UX2 3UXE 3UXF 3V0E 3V2I 3V38 3V46 3V4M 3V5C 3V66
3V68 3V7B 3VAV 3VBC 3VBJ 3VC5 3VEN 3VEU 3VFN 3VGL 3VHE 3VJ9 3VJZ 3VKA 3VKJ 3VL1 3VLV 3VM1 3VMN 3VMV 3VNB 3VNR 3VNY 3VOT 3VPB 3VPI 3VPQ 3VRD
3VRP 3VSY 3VTC 3VTU 3VTV 3VTX 3VU9 3VV1 3VV3 3VVY 3VWC 3VXG 3VXI 3VZ3 3VZH 3VZX 3W08 3W0K 3W0O 3W0Q 3W0S 3W15 3W2G 3W4S 3W52 3W7T 3W9K 3W9S
3WA7 3ZBO 3ZFN 3ZHF 3ZJH 3ZJI 3ZJL 3ZJM 3ZN6 3ZQI 3ZQK 3ZR0 3ZRI 3ZRV 3ZSU 3ZT9 3ZUI 3ZUZ 3ZWF 3ZWT 3ZX4 3ZYH 3ZYL 3ZYP 3ZYU 4A0D 4A2B 4A30
4A35 4A37 4A42 4A4Y 4A5K 4A5S 4A6Q 4A6U 4A6X 4A84 4A8T 4AC7 4ACY 4ADI 4ADN 4ADZ 4AE2 4AE4 4AEK 4AFV 4AGH 4AGP 4AHW 4AIW 4AJY 4AMF 4AML 4AO5
4AO6 4AO9 4AQ4 4AQJ 4AR9 4ARO 4ASC 4ASM 4ATM 4AUJ 4AVA 4AVD 4AWU 4AWY 4AXX 4AY0 4AZ7 4AZJ 4B0Z 4B1L 4B1V 4B2N 4B2O 4B4C 4B4P 4B72 4B89 4BA1
4BC3 4BCD 4BE3 4BGO 4BGP 4BH5 4BJ4 4BJT 4BJZ 4BK1 4BK3 4BOL 4BOU 4BQH 4BR5 4BR7 4BRA 4BRD 4BRK 4BWR 4D9B 4D9S 4DAM 4DB5 4DBC 4DDP 4DF3 4DH2
4DKK 4DL8 4DLN 4DM5 4DMC 4DMI 4DMK 4DMV 4DN7 4DNX 4DNY 4DO7 4DOI 4DOM 4DOY 4DQ6 4DQA 4DQD 4DRR 4DTH 4DUH 4DV8 4DWD 4DWN 4DY5 4DYQ 4DZO 4E15
4E1A 4E1B 4E2U 4E32 4E4T 4E5V 4E70 4EAD 4EBJ 4EBY 4ECF 4ECP 4EE7 4EF0 4EF8 4EHS 4EHU 4EKX 4EL6 4EMT 4EO0 4EPZ 4EQB 4EQG 4EQL 4ES5 4ES8 4ESQ
4ESU 4ESW 4EU3 4EUS 4EW1 4EW7 4EWR 4EX7 4EYS 4F0J 4F1J 4F3N 4F54 4F67 4F6T 4F8L 4FBS 4FCJ 4FCS 4FDB 4FDM 4FDV 4FE3 4FFL 4FGQ 4FKZ 4FNV 4FO7
4FOJ 4FP1 4FQS 4FR7 4FUS 4FVC 4FVG 4FVY 4FX5 4FYP 4G1J 4G1R 4G22 4G26 4G29 4G38 4G3V 4G48 4G4K 4G4P 4G4X 4G54 4G55 4G79 4G9M 4G9P 4G9Q 4GB5
4GBM 4GCY 4GD6 4GE3 4GEI 4GEK 4GF0 4GFT 4GGJ 4GH9 4GHG 4GHI 4GHN 4GKB 4GLQ 4GMM 4GNB 4GNF 4GNM 4GNO 4GNV 4GNY 4GOB 4GPV 4GQ6 4GT6 4GTA 4GUJ
4GVR 4GWK 4GXB 4GYF 4GYW 4GZJ 4GZK 4H04 4H08 4H0C 4H14 4H2G 4H41 4H59 4H5G 4H5S 4H6R 4H7U 4H7Y 4H8N 4HBA 4HBZ 4HD9 4HDK 4HF7 4HFS 4HGI 4HH3
4HHJ 4HHR 4HJF 4HKF 4HL1 4HLB 4HLC 4HMM 4HMU 4HMW 4HNG 4HPA 4HPU 4HQ1 4HRY 4HST 4HV3 4HW4 4HW6 4HXY 4HYQ 4HZ2 4HZ4 4HZA 4I1I 4I1K 4I3F 4I4V
4I56 4I6X 4I70 4I74 4I79 4I8I 4I90 4IA6 4IA9 4IAB 4IBN 4IDD 4IDH 4IHZ 4IIK 4IJI 4IJN 4IKC 4IKD 4IMQ 4IN9 4IND 4IO1 4IPB 4IPI 4IQM 4IQN 4IQY
4IR0 4IRG 4IRT 4IVA 4IVK 4IZB 4J27 4J2K 4J32 4J4H 4J4M 4J5F 4J5S 4J6O 4J79 4J7H 4J7N 4J8P 4J8S 4JA8 4JB3 4JBU 4JC1 4JDF 4JG3 4JGI 4JIF 4JKC
4JMD 4JN3 4JND 4JNF 4JPX 4JQF 4JQP 4JTF 4JTI 4JTJ 4JVB 4JWO 4JXC 4K05 4K0X 4K37 4K3F 4K3S 4K45 4K4K 4K60 4K73 4K8W 4KA2 4KAY 4KFN 4KH8 4KIL
4KK3 4KK4 4KK7 4KKV 4KM6 4KMY 4KNU 4KO1 4KP6 4KQ7 4KQC 4KSJ 4KUA 4KV1 4KVP 4KVS 4KWH 4KYQ 4KYV 4KZK 4L4G 4L5H 4L83 4L8E 4LB0 4LBA 4LC2 4LD6
4LFG 4LFY 4LG1 4LHR 4LLE 4LLS 4LMI 4LQB 4LRD 4LRU 4LSM 4LUI 4LW2 5CYT 5NUL 7ODC

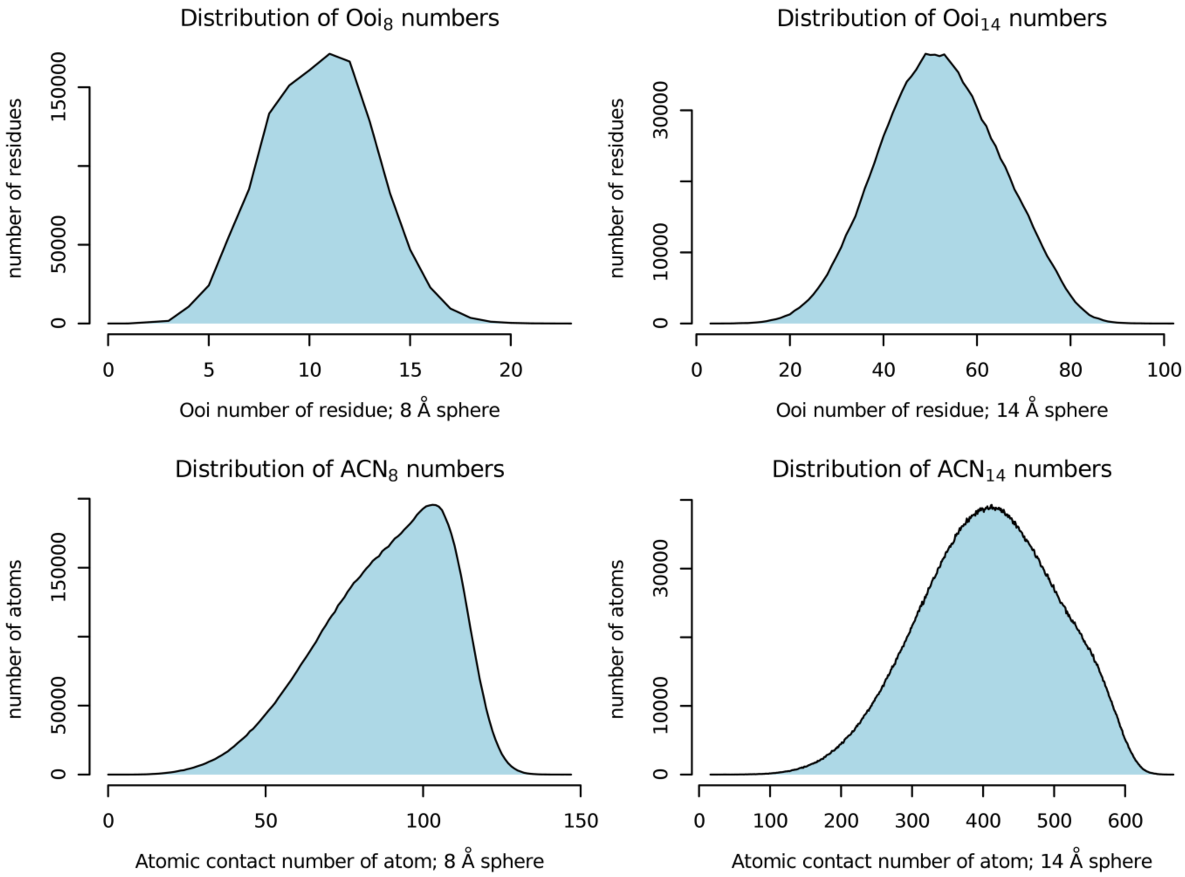

Figure S1 Distribution of Ooi numbers and atomic contact numbers (ACN) for radii of 8 Å and 14 Å for the 2,704 selected PDB structures. Note that both Ooi numbers and ACNs are discrete metrics; they can only take integer values.

1. Let the variable a denote a single atom.

2. Let the set A describe the set of all non-hydrogen protein atoms within the same PDB structure.

3. Choose a packing density function $P: A \rightarrow \mathbb{R}$ mapping every atom to a packing density. For this paper P was defined using atomic contact numbers with a range of 14 Å.

4. Let $B: A \rightarrow \mathbb{R}$ map every atom to its atomic B factor (atomic displacement parameter).

5. The function of average B factors returns the arithmetic mean of the atomic B factors of a set of atoms $X \subseteq A$:

$$\bar{B}(X) := \frac{\sum_{a \in X} B(a)}{|X|}$$

6. Define the packing density environments $E_n := \{a \in A \mid P(a) = n\}$ as sets, that contain all atoms with the packing density n as determined by P. The set of all nonempty E_n is a partition of A.

7. Similar packing density environments S_m are defined as sets of all atoms with a packing density *similar* to $m \in \mathbb{R}$:

$$S_m := \bigcup_{n \in sim(m)} E_n$$

8. Similarity can be defined by range, e.g. $sim(m) := \{x \in \mathbb{R} \mid m - \varepsilon \leq x \leq m + \varepsilon\}, \varepsilon \geq 0$ or by binning, e.g. $sim(m) := \left\{x \in \mathbb{R} \,\middle|\, \left\lfloor \frac{x}{z} \right\rfloor = \left\lfloor \frac{m}{z} \right\rfloor \right\}$ with a bin size $z > 0$. Here the latter definition was used with $z = 10$.

9. Define B_{Damage} of an atom a as the atomic B factor divided by the average B factor of all non-hydrogen protein atoms within the same PDB structure and with a similar packing density as a:

$$B_{Damage}(a) := \frac{B(a)}{\bar{B}\left(S_{P(a)}\right)}$$

Figure S2 Mathematical description of B_{Damage} calculation.

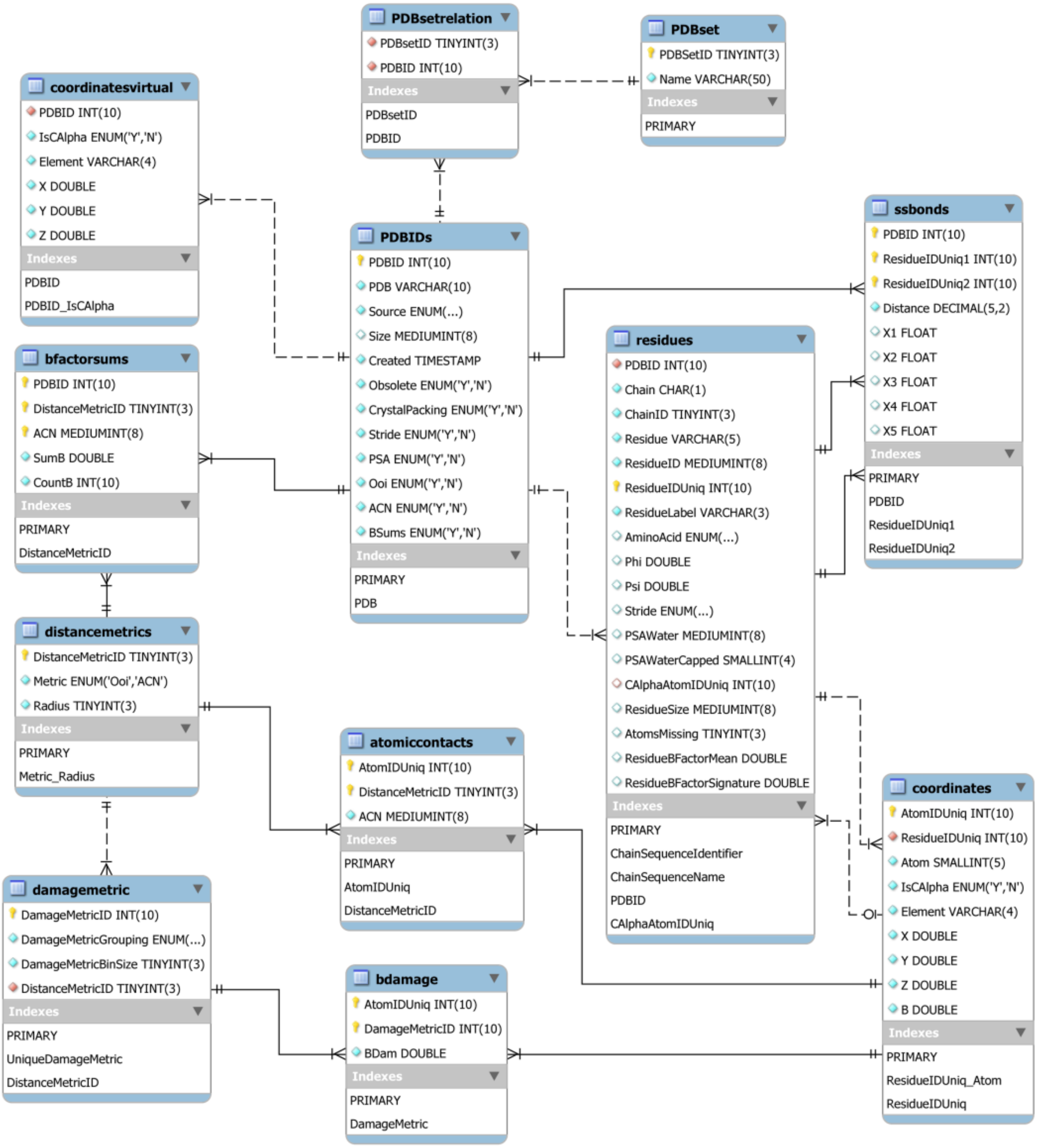

Figure S3 Entity-Relationship diagram of the relational database used for this investigation. Diagram prepared using MySQL Workbench 6.0 (http://mysqlworkbench.org/).

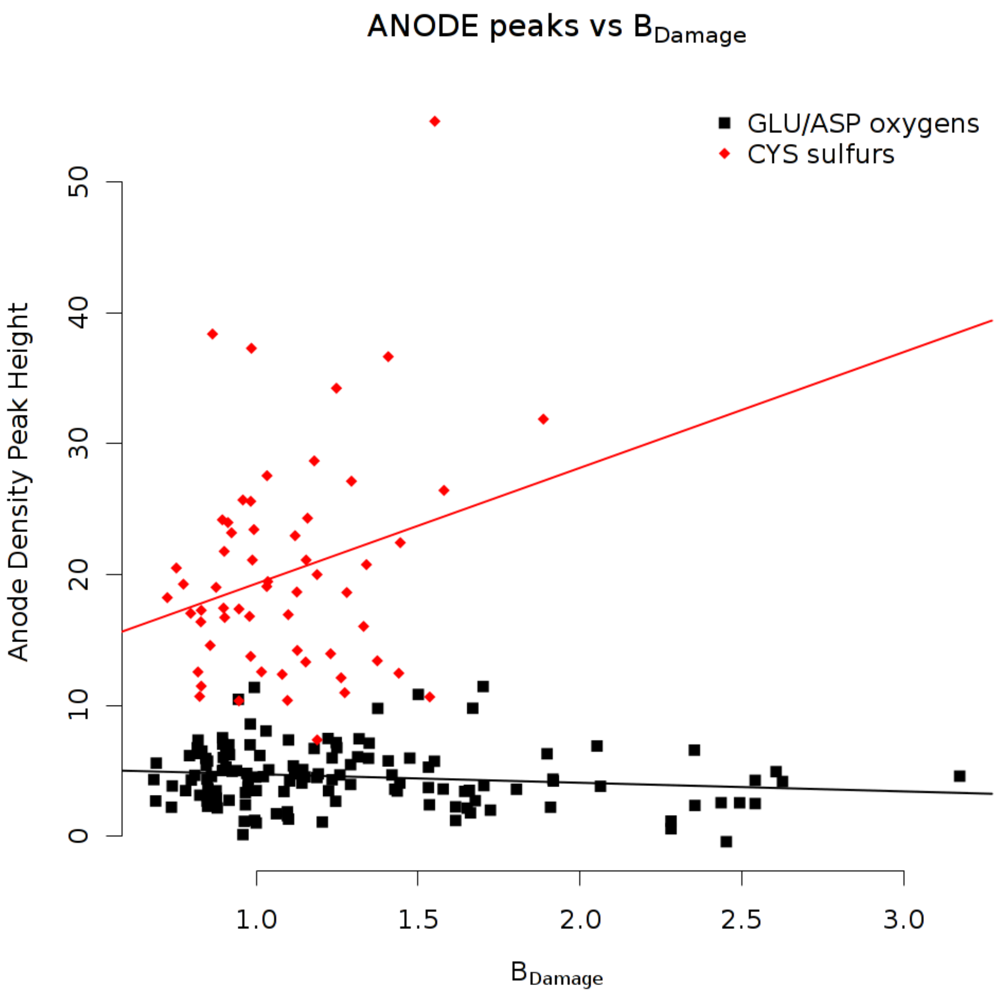

Figure S4 Peak heights of electron density difference maps around the cysteine sulfurs (red) and the terminal oxygens of glutamic and aspartic acids (black) plotted against their B_{Damage} values for the six Nanao protein datasets. Lines show least squares fits to the points. Peak heights were calculated using SHELXC (Sheldrick, 2010) and ANODE (Thorn & Sheldrick, 2011). B_{Damage} values were calculated for the relevant high-dose dataset.

There is some evidence for a correlation of high B_{Damage} values and strong electron density peak differences for cysteine sulfurs (CC=0.25, p=0.05). The apparent inverse correlation for GLU/ASP oxygens is not statistically significant (CC=-0.15, p=0.09).

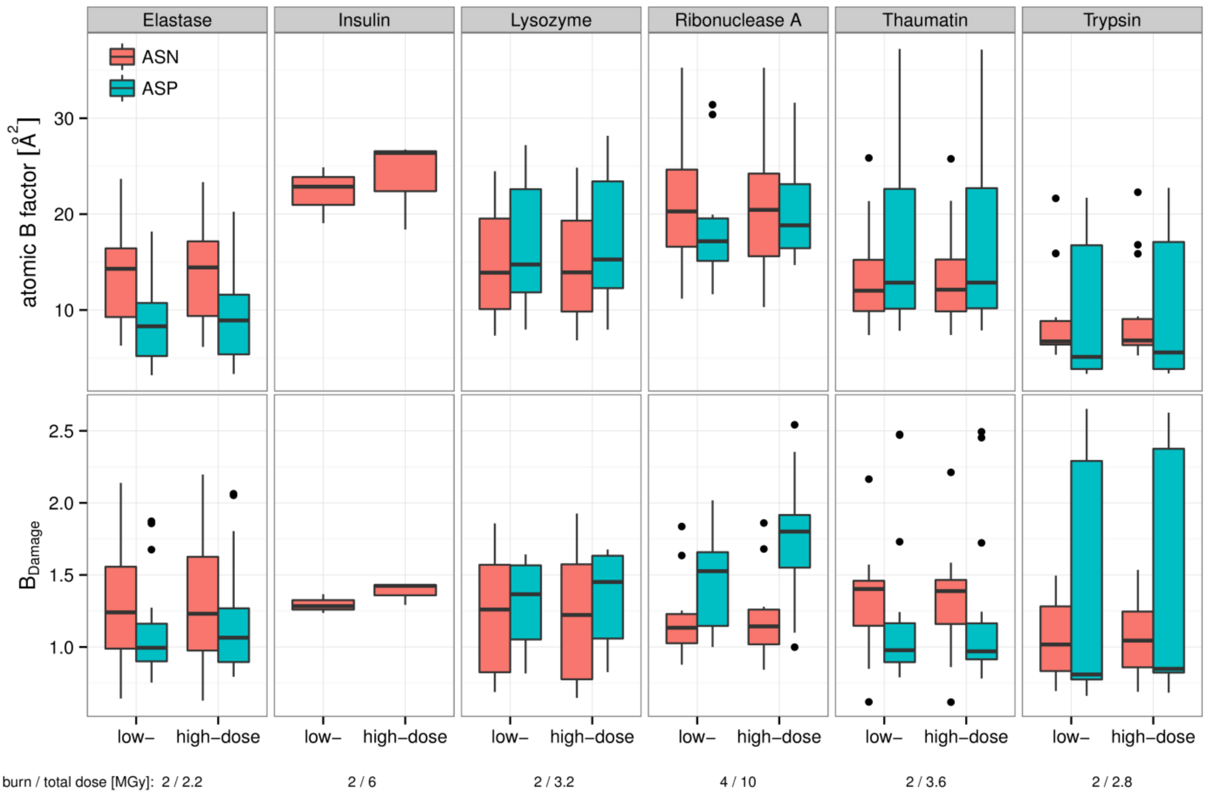

Figure S5 *B*-factor (**above**) and B_{Damage} (**below**) of ASP and ASN Oδ atoms of the six Nanao *et al.* (2005) proteins in two different dose states. The deterioration of the ASP side chain termini of ribonuclease A cannot be observed from the *B*-factor alone, but is clearly indicated by B_{Damage}. The ASN oxygens remain unaffected.

References

Sheldrick, G. M. (2010). Acta Cryst. D66, 479–485.

Thorn, A. & Sheldrick, G. M. (2011). J. Appl. Cryst. 44, 1285–1287.
